# Durable and High-Performance Thin-Film BHYb-Coated BZCYYb Bilayer Electrolytes for Proton-Conducting Reversible Solid Oxide Cells

**DOI:** 10.1021/acsami.3c04627

**Published:** 2023-06-28

**Authors:** Nicholas Kane, Zheyu Luo, Yucun Zhou, Yong Ding, Alex Weidenbach, Weilin Zhang, Meilin Liu

**Affiliations:** † School of Materials Science and Engineering, Georgia Institute of Technology, 771 Ferst Dr. NW, Atlanta, Georgia 30332-0245, United States; ‡ School of Electrical and Computer Engineering, Georgia Institute of Technology, 777 Atlantic Dr. NW, Atlanta, Georgia 30332-0250, United States

**Keywords:** electrolyte protection, bilayer electrolyte, reversible solid oxide cell, electrolysis, cosputtering

## Abstract

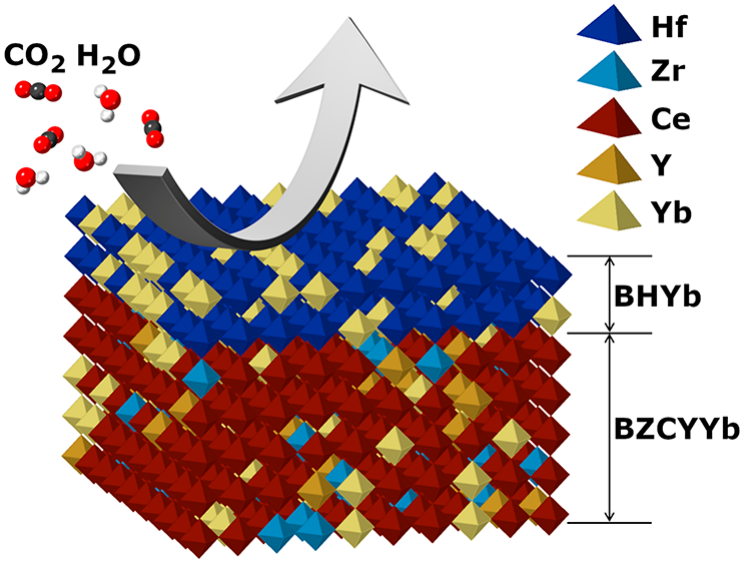

Proton-conducting reversible solid oxide cells are a promising technology for efficient conversion between electricity and chemical fuels, making them well-suited for the deployment of renewable energies and load leveling. However, state-of-the-art proton conductors are limited by an inherent trade-off between conductivity and stability. The bilayer electrolyte design bypasses this limitation by combining a highly conductive electrolyte backbone (e.g., BaZr_0.1_Ce_0.7_Y_0.1_Yb_0.1_O_3−δ_ (BZCYYb1711)) with a highly stable protection layer (e.g., BaHf_0.8_Yb_0.2_O_3−δ_ (BHYb82)). Here, a BHYb82-BZCYYb1711 bilayer electrolyte is developed, which dramatically enhances the chemical stability while maintaining high electrochemical performance. The dense and epitaxial BHYb82 protection layer effectively protects the BZCYYb1711 from degradation in contaminating atmospheres such as high concentrations of steam and CO_2_. When exposed to CO_2_ (3% H_2_O), the bilayer cell degrades at a rate of 0.4 to 1.1%/1000 h, which is much lower than the unmodified cells at 5.1 to 7.0%. The optimized BHYb82 thin-film coating adds negligible resistance to the BZCYYb1711 electrolyte while providing a greatly enhanced chemical stability. Bilayer-based single cells demonstrated state-of-the-art electrochemical performance, with a high peak power density of 1.22 W cm^–2^ in the fuel cell mode and −1.86 A cm^–2^ at 1.3 V in the electrolysis mode at 600 °C, while demonstrating excellent long-term stability.

## 1 Introduction

As the global energy industry phases out carbon-based fuels and incorporates intermittent renewable energy sources, there is a large demand for efficient grid-level storage to bridge the gap between where and when electricity is produced and used. Reversible solid oxide cells are one promising technology for grid-level storage as they can efficiently switch between electricity generation from chemical fuels and fuel production from electrolysis of water and CO_2_. Recently, proton-conducting reversible solid oxide cells (P-ReSOCs) have attracted much attention due to their unique advantages. In P-ReSOCs, the steam is applied to the air electrode, as opposed to oxygen ion conducting cells, where the steam is applied to the fuel electrode. This eliminates the potential for Ni oxidation in the fuel electrode and the need for downstream purification to remove water, simplifying the system.^1^ However, this exposes the air electrode side of the electrolyte to high concentrations of water. BaZr_0.1_Ce_0.7_Y_0.1_Yb_0.1_O_3-δ_ (BZCYYb1711) is a state-of-the-art proton conductor with excellent ionic conductivity and a high protonic transference number; however, it degrades in high concentrations of water and CO_2_.^2−7^ The stability of BaZr_*x*_Ce_0.8–*x*_Y_0.1_Yb_0.1_O_3−δ_ (BZCYYb) can be improved by increasing the zirconium content; however, this decreases the conductivity, leading to a decrease in performance.^8,9^ Thermodynamic calculations and experimental studies have shown that zirconate-, hafnate-, and titanate-based perovskite-type electrolytes are more stable.^8−12^ For example, BaZr_0.4_Ce_0.4_Y_0.1_Yb_0.1_O_3−δ_ (BZCYYb4411), with a higher content of Zr, offers superior stability compared to BZCYYb1711, but its conductivity is significantly diminished.^13−17^ Thus, there is an inherent trade-off between conductivity and stability within the BZCYYb system.^18−20^

One approach to improving stability while maintaining low resistance is to create a bilayer electrolyte^21,22^ consisting of a highly stable electrolyte (e.g., BaZr_0.8_Y_0.2_O_3−δ_) layer and a highly conductive electrolyte (e.g., BaCe_0.8_Y_0.2_O_3−δ_) layer.^23−27^ The bilayer electrolytes offer superior stability against high concentrations of contaminants; however, the overall performance is typically decreased due to the high ohmic resistance of the relatively thick (e.g., 1–30 μm) stability layer.^21,28^ Additionally, the performance of the bilayer electrolytes is limited by the quality of the film. Porosity from suspension coating or dry pressing techniques greatly increase the ohmic resistance of the protection layer.^29,30^ In comparison, thin-film deposition techniques, such as pulsed laser deposition and sputtering, offer superior quality films and allow for the reduction of the overall thickness.^31,32^ Furthermore, they do not require high-temperature sintering, which reduces interdiffusion between the layers.^33^ This is advantageous as dissolution of the protection film into the bulk will decrease both the bulk conductivity and the chemical stability of the surface, while better chemical stability is offered by a distinct stability phase on the surface.

Recently, a new class of proton conducting electrolytes based on BaHfO_3_ was reported, which offer superior stability compared to the conventional barium zirconate-based systems.^34^ However, the conductivity of barium hafnate-based systems remains lower than that of barium cerate-based systems. Here, we report the fabrication of a BaHf_0.8_Yb_0.2_O_3−δ_ (BHYb82) electrolyte protection layer via cosputtering to form a BHYb82-BZCYYb1711 (BHYb-BZCYYb) bilayer electrolyte. The 110 nm BHYb82 protection layer greatly enhances the stability of the electrolyte while having little effect on the electrochemical performance of the cell. The bilayer electrolytes are stable in pure CO_2_ as well as high concentrations of H_2_O and effectively bypass the typical trade-off between conductivity and stability, while achieving state-of-the-art electrochemical performance.

## 2 Results and Discussion

### 2.1 Electrolyte Properties

**Figure 1:**
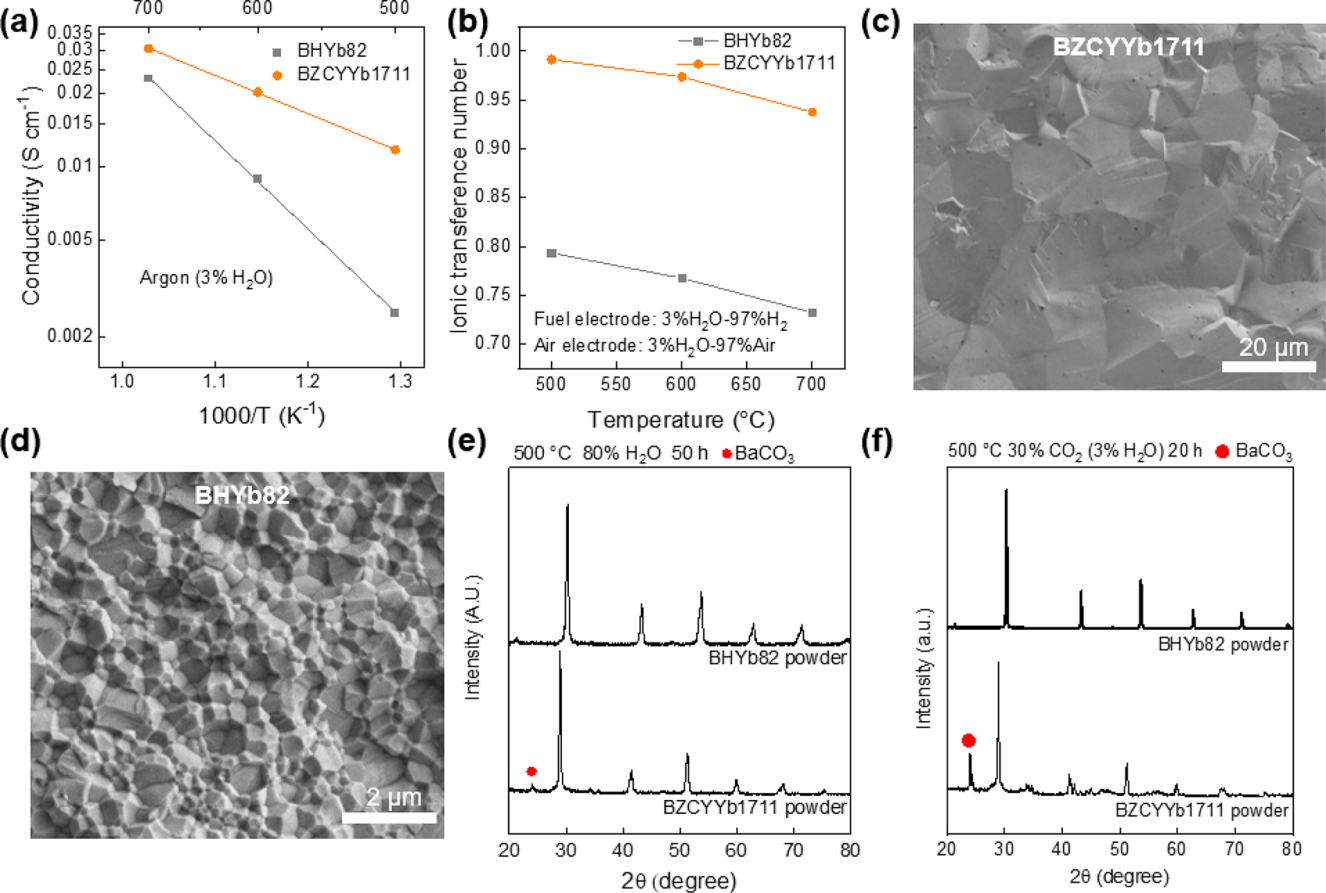
Comparison of the properties of BZCYYb1711 and BHYb82. (a) Average conductivity as a function of temperature. (b) Ionic transference number as a function of temperature with H_2_ (3% H_2_O) and air (3% H_2_O) on either side. SEM images of sintered (c) BZCYYb1711 and (d) BHYb82 at 1400 °C for 5 h. XRD patterns of BZCYYb1711 and BHYb82 powder exposed to (e) 80% H_2_O balance Ar at 500 °C for 50 h and (f) 30% CO_2_ balance Ar for 20 h at 500 °C.

Figure 1 compares important electrolyte properties between BZCYYb1711 and BHYb82, including conductivity, ionic transference number, sinterability, and chemical stability. The data show that the electrochemical properties of BZCYYb1711, such as conductivity and ionic transference number, are far superior to that of BHYb82. For example, the conductivity of BZCYYb1711 at 500 °C is 0.012 S cm^–1^, compared to 0.0025 S cm^–1^ for BHYb82, an increase of five times (see Figure 1a). Additionally, the ionic transference number of BZCYYb1711 at 500 °C is 0.99, compared to 0.79 for BHYb82 (see Figure 1b). Furthermore, the sintereability of BHYb82 is low, as evident by the much smaller BHYb82 grain size as compared to BZCYYb1711 after sintering at 1400 °C for 5 h (see Figure 1c,d). From this comparison, it is clear that the properties of BHYb82 are not sufficient for use as an electrolyte material, as the low conductivity would result in large ohmic losses and the low ionic transference number would result in significant electronic leakage, especially during electrolysis. The poor sinterability of BHYb82 leads to a higher grain boundary density, which in turn reduces the conductivity of the electrolyte.^35^ Additionally, a higher sintering temperature is required for densification, which increases barium evaporation and nickel diffusion from the fuel electrode, ultimately changing the composition of the electrolyte.^36^ In contrast, the chemical stability of BHYb82 is superior to that of BZCYYb1711, showing no degradation in the presence of H_2_O and CO_2_. Figure 1e,f shows the degradation of BZCYYb1711 via the formation of BaCO_3_ while no degradation is seen for BHYb82. These data demonstrate the inherent trade-off in the barium cerate–barium hafnate system and justify the need for a bilayer electrolyte design, which can achieve both high conductivity and chemical stability. In order to verify the chemical compatibility of BHYb82 and the PBCC electrode, the two powders were mixed and fired at 1000 °C for 4 h. XRD patterns of the mixture, as shown in Figure S1, verify that BHYb82 and PBCC remain as two distinct phases, with no minority phases present.

### 2.2 Thin-Film Deposition and Analysis

The deposition of BHYb82 films was extensively investigated to optimize the composition and morphology of the film. Initial BHYb82 films were deposited via single target RF magnetron sputtering of BHYb82. The films showed poor phase formation and severe barium deficiency, as measured by X-ray diffraction (XRD) and energy dispersive X-ray spectroscopy (EDX) (Figure S2 and Table S1). To achieve the proper stoichiometry, BHYb82 thin films were deposited via cosputtering, using a Ba metal target to adjust the A-site to B-site ratio (see Figure S3).

Table S2 shows an EDX analysis of the BHYb82 film. Because of the penetration depth of EDX and the similar elemental compositions of BHYb82 and BZCYYb1711, a silver substrate was utilized for the EDX analysis to allow for easier quantification of the film composition. The analysis shows that the BHYb82 film is very close to that of the ideal composition. Critically, the A-site (Ba) to B-site (Hf and Yb) ratio is ideal at 1. The film is slightly rich in Yb, leading to a slightly lower Ba:Hf ratio of 1.21, as compared to the ideal ratio of 1.25 for BHYb82; however, this does not significantly affect the properties of the film.^37^ The EDX data verify the cosputtering technique achieved the proper stoichiometry.

**Figure 2:**
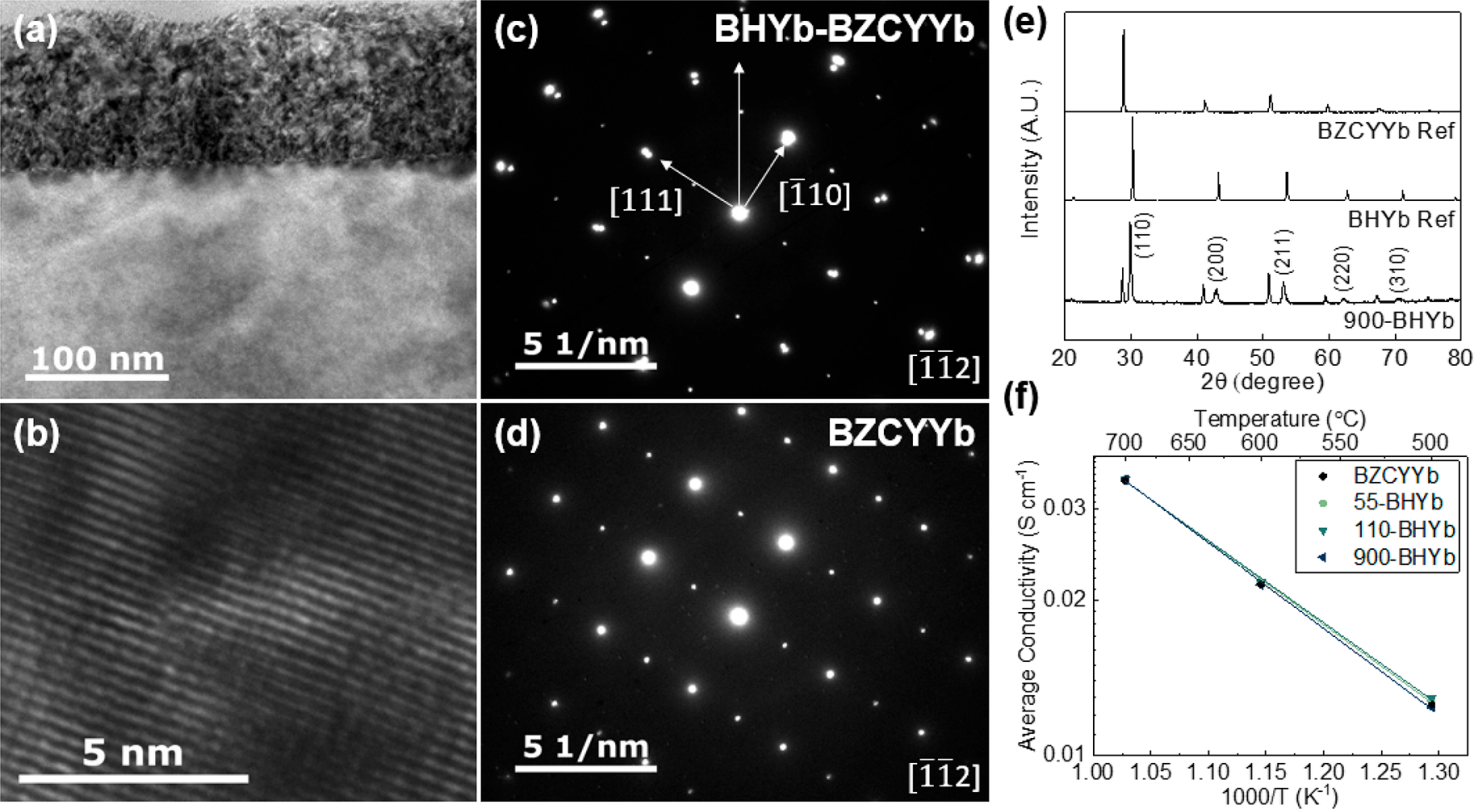
Optimized BHYb-BZCYYb bilayer electrolytes. (a, b) HRTEM images of the 110-BHYb bilayer electrolyte. SAED patterns of the (c) 110-BHYb bilayer and (d) BZCYYb1711 substrate with the direction of the interface indicated. (e) XRD patterns of BZCYYb1711 and BHYb82 references and 900-BHYb. (f) Average conductivity as a function of temperature for unmodified BZCYYb1711 and BHYb-BZCYYb bilayer electrolytes with various BHYb82 thicknesses from 55 to 900 nm.

With proper film stoichiometry, the phase, density, and morphology greatly improved. Three different film thicknesses were investigated: 15, 55, and 110 nm. The maximum thickness was limited by the need to maintain a low ohmic resistance. Figure 2 shows the analysis of the optimized BHYb-BZCYYb bilayer electrolytes with a BHYb82 thickness of 110 nm. Bilayer electrolytes are denoted with the protection layer thickness in nanometers, i.e., 110-BHYb. The high-resolution transmission electron microscope (HRTEM) image shown in Figure 2a displays defects and strain in the BHYb82 as seen from the darkness in the film. The large amount of defects present is likely associated with the low-angle grain boundaries between grain columns seen in Figure S4a. Additionally, dislocations likely result from the stress in the lattice due to the lattice mismatch between the BHYb82 and BZCYYb1711. Point defects and trapped Ar may also be present, resulting from the sputtering process. Additionally, the BHYb82 film is continuous and adheres well over the entire area of the cell, as shown in Figure S4b. The sputtered film conforms to the surface topography of the BZCYYb1711 half-cell, generating a conformal coating/bilayer electrolyte. No large voids or delaminations are seen in the film. Faceting of the film resulting from self-shadowing is present where the local surface normal of the half-cell significantly deviates from the overall surface normal (Figures S5 and S6). Figure 2b shows an HRTEM image of the bilayer interface. The image shows an epitaxial interface, with strain extending into the BZCYYb1711 substrate, resulting from the lattice mismatch. Selective area electron diffraction (SAED) patterns are shown in Figure 2c,d, which confirm that the BHYb82 film and BZCYYb1711 substrate are epitaxially orientated with a slight lattice mismatch. Additionally, a second location was analyzed with HRTEM, as shown in Figure S7, which confirmed that the epitaxial growth is consistent across the cell. Considering the BZCYYb1711 substrate is polycrystalline and randomly orientated, the epitaxial BHYb82 film must also contain orientated domains aligning with the polycrystalline substrate. Thus, on the micrometer scale, the film is polycrystalline, containing epitaxial columnar domains, which are each aligned to a specific grain in the polycrystalline substrate on which they grew. This is consistent with the bulk X-ray diffraction data (see Figure 2e) which show that the BHYb82 film does not have a preferred orientation. Additionally, the XRD analysis shows two distinct phases, BZCYYb1711 and BHYb82, with no indication of a solid solution or impurity phases. To further investigate the possibility of a solid solution between BHYb82 and BZCYYb1711, the two powders were mixed in an equal ratio and fired at 1000 °C for 4 h. The XRD patterns in Figure S8 show no reaction between the two materials, which further confirms that the bilayer maintains two distinct phases. Additionally, Figure 2e shows a small peak shift for the BHYb82 film compared with the BHYb82 powder reference. The (220) peak was measured at 29.86° for the BHYb82 film as compared to 30.24° for the reference, corresponding to a decrease of 0.38°. This is likely caused by straining of the film due to the lattice mismatch between the BZCYYb1711 substrate and BHYb82 film, which will increase the atomic spacing in the film due to the higher lattice constant of BZCYYb1711 as compared to BHYb82. As shown in Figure S9, the phase of the film does not change after annealing at 950 °C for 2 h, which is required for air electrode fabrication. Figure 2f shows the average conductivity as a function of temperature for various bilayer electrolytes and unmodified BZCYYb1711 with an electrolyte-supported cell configuration of Ag|BHYb82(if applicable)|BZCYYb1711|Ag. To enhance the contribution of the film to the overall resistance, a thick 900 nm BHYb82 electrolyte protection layer was fabricated. The average conductivity of 900-BHYb was comparable to that of unmodified BZCYYb1711 at all tested temperatures. Thus, it is concluded that the conductivity of the film is sufficient and does not increase the resistance of the cell.

**Figure 3:**
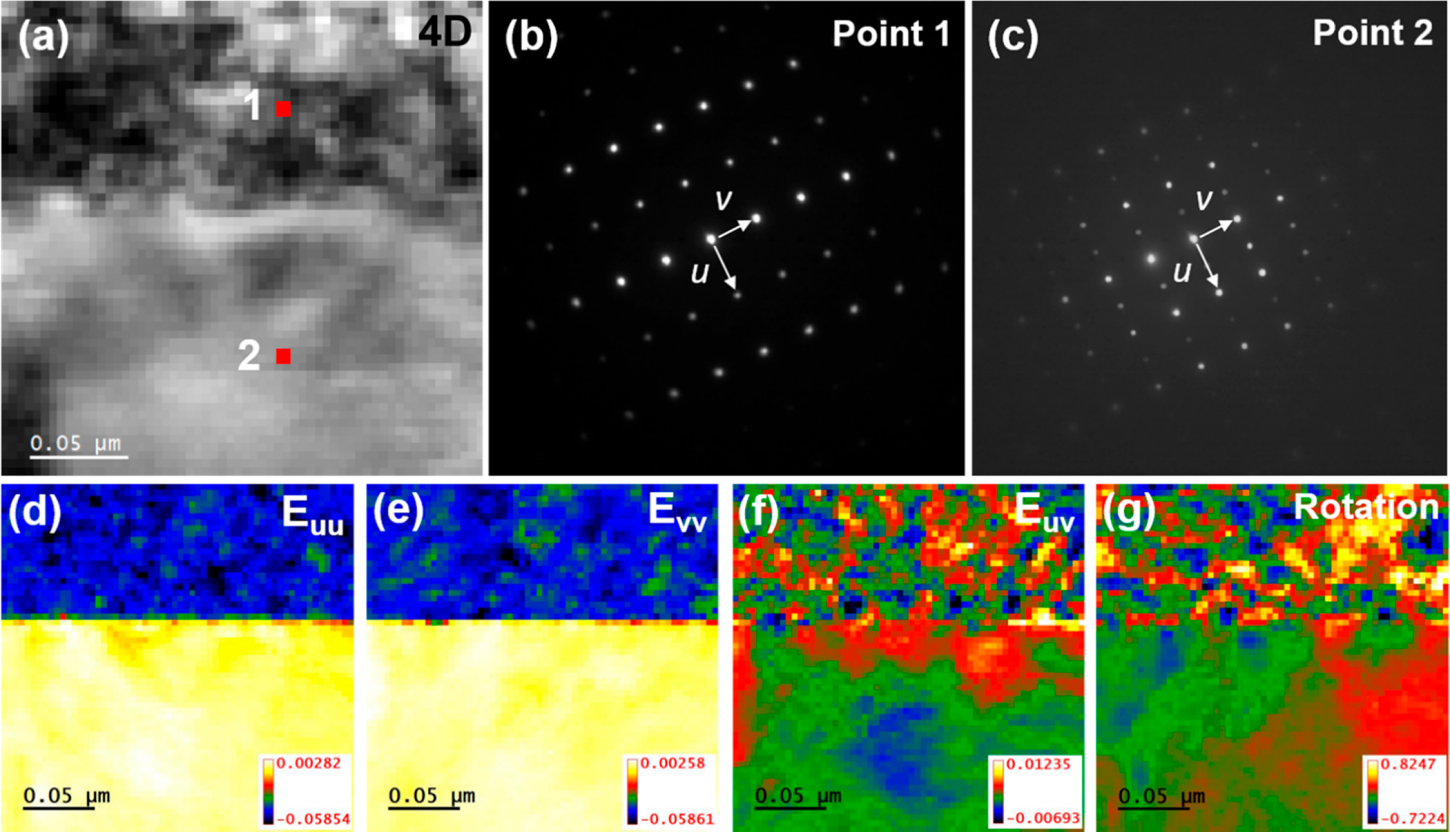
4D STEM analysis of the 110-BHYb bilayer electrolyte. (a) 4D STEM image. Electron diffraction patterns from (a) point 1 and (b) point 2, as noted in the 4D STEM image. Stain and orientation maps showing the (d) in-plane strain (*E*_uu_), (e) out-of-plane strain (*E*_vv_), (f) shear strain (*E*_uv_), and (g) lattice rotation.

Figure 3 shows a 4D STEM analysis of the 110-BHYb bilayer. For the 4D STEM image shown in Figure 3a, each pixel (approximately 4 nm × 4 nm) represents the location of an SAED pattern. Figure 3b,c shows representative SAED patterns from points 1 and 2 as denoted in Figure 3a. These diffraction patterns confirm the phase of the film. Figure 3d,e shows the in-plane and out-of-plane strain maps, which highlight the difference in lattice parameters for the two materials. As shown, the lattice constant of the BHYb82 film is approximately 5.8% smaller than that of the BZCYYb1711 substrate. The shear strain map shown in Figure 3f displays shear strains around the interface and extending 200 nm into the BZCYYb1711 substrate. Additionally, the film is strained throughout the entire thickness, especially at the interface. Relaxed regions near the interface are likely the result of dislocations releasing stress, as shown in Figure 2b. Figure 3g shows the lattice rotation map within the bilayer, indicating a small rotation of grains within the film.

### 2.3 Chemical Stability

**Figure 4:**
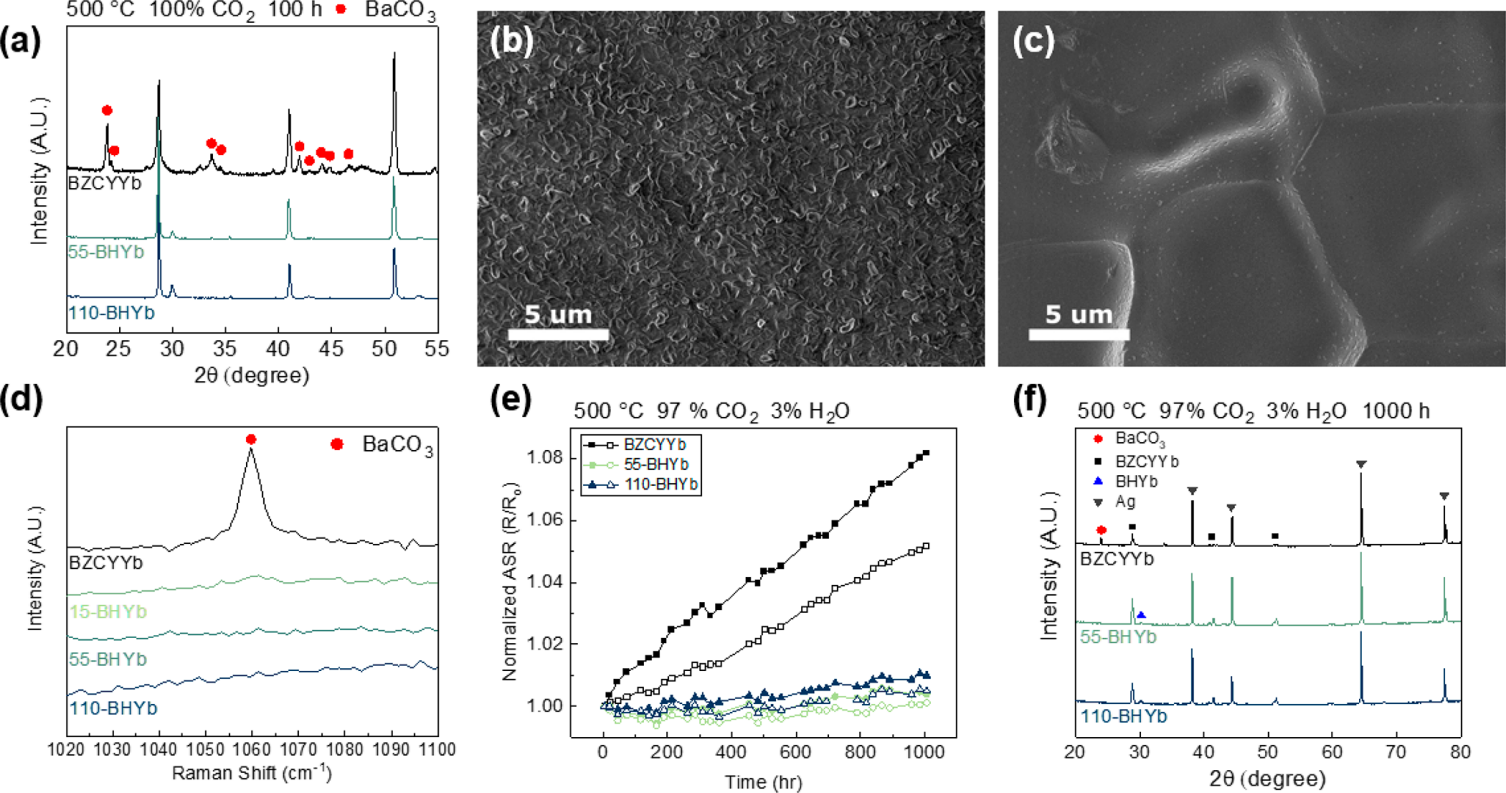
Chemical stability testing of the bilayer electrolytes. (a) XRD analysis of the unmodified BZCYYb1711 and BHYb-BZCYYb bilayer electrolytes. SEM images of the surface of (b) unmodified BZCYYb1711 and (c) 110-BHYb after exposure to 100% CO_2_ for 100 h at 500 °C. (d) Raman spectra of unmodified BZCYYb1711 and BHYb-BZCYYb bilayer electrolytes after exposure to 100% CO_2_ for 100 h at 500 °C. (e) Normalized area specific resistance as a function of time for bare BZCYYb1711 and various thicknesses of BHYb-BZCYYb bilayer electrolytes in 97% CO_2_ with 3% H_2_O at 500 °C. The cell configuration is Ag|BHYb82|BZCYYb1711|BHYb82|Ag. (f) XRD patterns of the bare BZCYYb1711, 55-BHYb, and 110-BHYb electrolytes after exposure to 97% CO_2_ with 3% H_2_O at 500 °C for 1000 h.

In order to investigate the chemical stability of the bilayers, we exposed the bilayer electrolytes to 100% CO_2_ for 100 h at 500 °C. Figure 4 shows the chemical stability analysis of the unmodified BZCYYb1711 and BHYb-BZCYYb bilayer electrolytes. The XRD analysis in Figure 4a shows that the degradation of the unmodified BZCYYb1711 is very severe, with the formation of large amounts of BaCO_3_. In contrast, the 55-BHYb and 110-BHYb bilayer electrolytes are stable, with no BaCO_3_ formation detectable by XRD. The SEM images confirm the severe degradation of unmodified BCZYYb1711. As seen in Figure 4b, the surface is completely covered by BaCO_3_, thick enough to obscure the grain structure of the electrolyte. In contrast, only minor BaCO_3_ formation is observed on the 110-BHYb bilayer electrolyte, as seen in Figure 4c. Additional SEM images are shown in Figure S10. Small facets are seen emerging from the film; however, these are typically observed in the pristine bilayer surface (see Figure S5b). Additionally, small degradation particles are observed on the grain boundary and nonflat regions of the cell. Because of the small quantity and size of the particles, it is difficult to identify their phase and composition; however, they are most likely related to the degradation of the electrolyte. The presence of these particles indicates potential defects in the film, which allow slight penetration of the film. As discussed previously, the rough topography of the sintered half-cell surface reduces the quality of the film in these regions. At these defects, slight degradation is observed. However, as no BaCO_3_ was detected by XRD or Raman spectroscopy (Figures 4d and S11), the extent of degradation was insignificant. The CO_2_ stability study verifies the stability of the bilayer electrolyte. In order to further investigate the effect of cell topography, 110-BHYb was prepared on both as-sintered and polished BZCYYb1711 pellets. As shown in Figure S12, the typical film defects are not observed and no degradation is present, attributed to the increased flatness from the polishing. In contrast, degradation is present around the nonflat regions on the as-sintered cell. The enhanced stability is present in bilayers, with a stability layer as thin as 15 nm. This indicates that a thick layer is not required to protect the electrolyte, which is beneficial for the electrochemical performance of the cell. Minimizing the thickness of the less conductive layer allows for a minimal impact on the ohmic resistance and thus the electrochemical performance.

The normalized area specific resistance (ASR) of the bilayer electrolytes was also measured in CO_2_ with 3% H_2_O to verify the impact of degradation on the electrochemical performance, as shown in Figure 4e. The electrolyte protection layer was applied to both sides of the pellet for complete protection against the contaminants. The data show that the resistance of the bilayer electrolytes is stable for 1000 h, with very little degradation measured. In contrast, the unmodified BZCYYb1711 electrolytes experience significant degradation, continuously increasing their resistance over the entire test. The degradation rates calculated from linear regression fitting are 5.1 and 7.0%/1000 h for the bare BZCYYb1711, compared to 0.4 to 1.1%/1000 h for the 55-BHYb and 110-BHYb bilayers. The increase in resistance is attributed to the degradation of BZCYYb1711 and formation of insulating barium carbonate and other oxides, as shown previously with XRD and Raman analysis. Additionally, no significant difference is observed between the 55-BHYb and 110-BHYb, indicating that the 55 nm film provides adequate protection. The slight degradation of the bilayer cells is attributed to the defected regions of the film on the cell. For comparison, the conductivity of stable BZCYYb4411 at 500 °C is around 8 × 10^–3^ S/m, which is about 60% of the bilayer electrolytes (see Figure S13). These data verify the excellent performance of the bilayer electrolytes, achieving the high conductivity of BZCYYb1711 while remaining stable in wet CO_2_. Figures 4f and S14 show the XRD analysis of the cells after the 1000 h stability test in CO_2_ and H_2_O. The data show significant degradation of BZCYYb1711 to form barium carbonate, while no observable degradation is detected for 55-BHYb and 110-BHYb. Figure S15 shows the surface of the cells after the 1000 h stability test. Consistent with Figure 4, the bare BZCYYb1711 surface shows severe degradation, while the 55-BHYb and 110-BHYb films greatly reduced the degradation. Contaminants are observed around the nonflat regions of the cell, while the flat interior of the grains are largely protected. This slight degradation is likely the cause of the small degradation rate seen in Figure 4e. Murphy et al. first demonstrated the improved chemical stability of BaHfO_3_-based compositions as compared to BaZrO_3_-based compositions in bulk electrolytes.^34^ For the bilayer cells, the BHYb82 provides improved stability as it protects BZCYYb1711 from degradation in the contaminating atmospheres. The dense, relatively inert BHYb82 prevents the CO_2_ from reaching and reacting with the BZCYYb1711, leading to improved stability.

### 2.4 Electrochemical Performance

**Figure 5:**
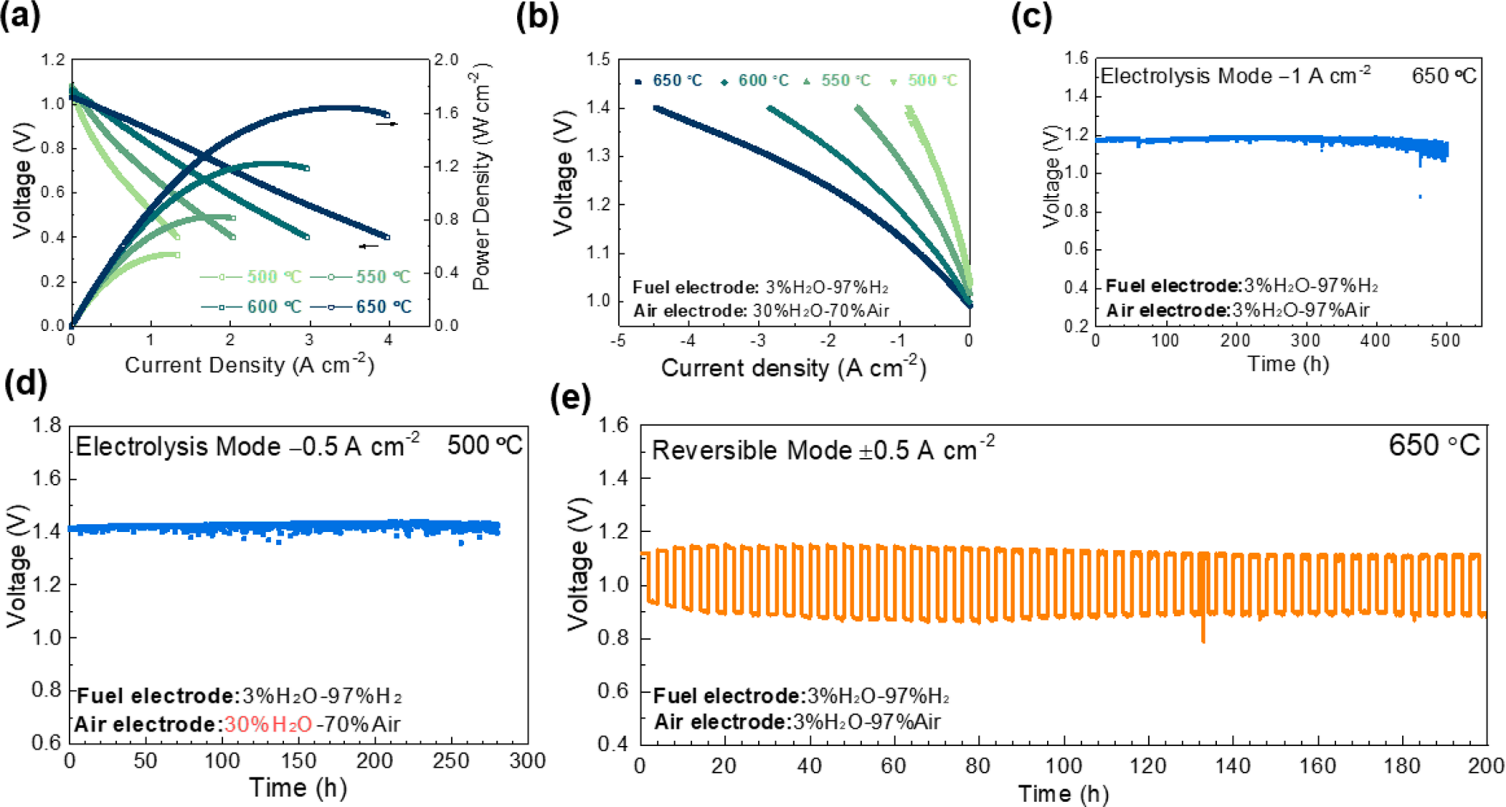
Electrochemical performance of PBCC|BHYb82|BZCYYb1711|Ni-BZCYYb1711 fuel-electrode-supported cells. (a) Typical *I*–*V*–*P* curves in the fuel cell mode with H_2_ (3% H_2_O) in the fuel electrode and ambient air as the oxidant from 500 to 650 °C. (b) Typical *I*–*V* curves in the electrolysis mode with H_2_ (3% H_2_O) in the fuel electrode and 30% H_2_O balanced air in the air electrode. Long-term stability in the electrolysis mode with (c) 3% H_2_O and (d) 30% H_2_O balance air in the air electrode. (e) Reversible operation at 600 °C at a current density of ±0.5 A cm^–2^ switching between modes every 2 h.

Figure 5 shows the typical electrochemical performance of the bilayer-based cells with a configuration of PrBa_0.8_Ca_0.2_Co_2_O_5+δ_ (PBCC)|BHYb82|BZCYYb1711|Ni-BZCYYb1711, an active electrode area of 0.28 cm^2^, and a BZCYYb1711 electrolyte thickness of 10 μm. The BHYb82 layer thickness was selected to be 110 nm from the chemical stability studies as this thickness provides excellent stability without affecting the electrochemical performance. SEM images of the cross section of the bilayer-based cells, seen in Figure S16, show the BHYb82 layer and PBCC electrode are well adhered. As shown in Figure 5a, the peak power densities of the bilayer-based cells were 1.64, 1.22, 0.82, and 0.54 W cm^–2^ at 650, 600, 550, and 500 °C, respectively. In comparison, PBCC|BZCYYb1711|Ni-BZCYYb1711 cells reported by Zhou et al.,^38^ which are nearly identical cells (including the same BZCYYb1711 electrolyte thickness of 10 μm) without the BHYb82 protection layer, achieved 1.58, 1.06, and 0.66 W cm^–2^ at 650, 600, and 550 °C. Thus, the performance of the bilayer-based cells is comparable to that of the unmodified cells and demonstrated state-of-the-art peak power densities exceeding previously reported P-ReSOCs.^39^ Additionally, the peak power density exceeds that of similar bilayer-based P-ReSOCs, as shown in Table 1. The average of the peak power density of the five cells is shown in Figure S17, demonstrating a reproducible performance. Additionally, the Nyquist plots shown in Figure S18 indicate no increase in ohmic resistance as compared to the unmodified cells. For example, the ohmic resistance of the bilayer-based cells was 0.075 Ω cm^2^ at 650 °C as compared to 0.080 Ω cm^2^ for the unmodified cell.^38^Figure 5b shows the typical *I*–*V* curves in the electrolysis mode when the cell is exposed to H_2_ (3% H_2_O) and 30% H_2_O balance air at 500 to 650 °C. The bilayer-based cells achieved current densities of −2.84, −1.86, −1.03, and −0.56 A cm^–2^ at 1.3 V and 650, 600, 550, and 500 °C, respectively, which are among the highest records reported to date.^40,41^ Additionally, stable operation in the electrolysis mode at −1 A cm^–2^ and 600 °C is demonstrated for up to 500 h in 3% H_2_O balance air (Figure 5c) as well as 280 h in 30% H_2_O balance air at 500 °C (Figure 5d). After stability testing for 500 h, no delamination or degradation of the bilayer is present, as shown in Figure S19. Finally, excellent reversibility is demonstrated for 200 h at a current density of 0.5 A cm^–2^, switching between modes every 2 h. These data demonstrate that the application of the electrolyte protection layer did not affect the performance of the cell while greatly enhancing the stability.

**Table 1:** Comparison of Recent Proton Conducting SOCs with Bilayer Electrolytes

bilayer electrolyte	thickness (μm)	fuel electrode	air electrode	fabrication method	peak power density	ref
BaCe_0.8_Y_0.2_O_3−δ_/BaZr_0.7_Pr_0.1._Y_0.2_O_3−δ_	28, 3.3	NiO-BaCe_0.8_Y_0.2_O_3−δ_	Sm_0.5_Sr_0.5_CoO_3−δ_-Ce_0.8_Sm_0.2_O_3−δ_	PLD	177 mW at 650 °C	(23)
BaCe_0.8_Y_0.2_O_3−δ_/BaZr_0.8_Y_0.2_O_3−δ_	29, 0.7–3.6	NiO-BaZr_0.1_Ce_0.7_Y_0.2_O_3−δ_	Sm_0.5_Sr_0.5_CoO_3−δ_-Ce_0.8_Sm_0.2_O_3−δ_	PLD	301 mW at 650 °C	(26)
BaCe_0.85_Y_0.15_O_3−δ_/BaZr_0.85_Y_0.15_O_3−δ_	15, 15	NiO-BaCe_0.85_Y_0.15_O_3−δ_	La_0.6_Sr_0.4_Co_0.2_Fe_0.8_O_3−δ_	drop coating	30 mW at 600 °C	(42)
BaCe_0.9_Y_0.1_O_3−δ_/BaZr_0.85_Y_0.15_O_3−δ_	10, 10	NiO-BaZr_0.85_Y_0.15_O_3−δ_	Ba_0.5_Sr_0.5_Co_0.8_Fe_0.2_O_3−δ_-BaCe_0.9_Y_0.1_O_3−δ_	screen printing	502 mW at 600 °C	(43)
BaCe_0.8_Y_0.2_O_3−δ_/BaZr_0.8_Y_0.2_O_3−δ_	1000, 1	Pt	Pt	PLD	22 mW at 700 °C	(27)
BaCe_0.8_Y_0.2_O_3−δ_/BaZr_0.4_Ce_0.4_Y_0.2_O_3−δ_	23, 35	NiO-BaZr_0.4_Ce_0.4_Y_0.2_O_3−δ_	Ba_0.5_Sr_0.5_Co_0.8_Fe_0.2_O_3−δ_	co-pressing	205 mW at 650 °C	(24)
BaZr_0.1_Ce_0.7_Y_0.2_O_3−δ_/BaZr_0.8_Y_0.2_O_3−δ_	5, 3	NiO-BaZr_0.1_Ce_0.7_Y_0.2_O_3−δ_	La_0.6_Sr_0.4_Co_0.2_Fe_0.8_O_3−δ_-BaZr_0.8_Y_0.2_O_3−δ_	suspension coating	131 mW at 650 °C	(25)
BaZr_0.7_Pr_0.1._Y_0.2_O_3−δ_/BaCe_0.8_Y_0.2_O_3−δ_/BaZr_0.7_Pr_0.1._Y_0.2_O_3−δ_	10, 45, 10	NiO-BaZr_0.7_Pr_0.1._Y_0.2_O_3−δ_	La_0.6_Sr_0.4_Co_0.2_Fe_0.8_O_3−δ_-BaZr_0.7_Pr_0.1._Y_0.2_O_3−δ_	co-pressing	103 mW at 650 °C	(10)
BaZr_0.1_Ce_0.7_Y_0.1_Yb_0.1_O_3−δ_/BaHf_0.8_Y_0.2_O_3−δ_	10, 0.1–0.9	NiO-BaZr_0.1_Ce_0.7_Y_0.1_Yb_0.1_O_3−δ_	PrBa_0.8_Ca_0.2_Co_2_O_5+δ_	sputtering	1640 mW at 650 °C	this work

## 3 Conclusion

A BHYb-BZCYYb bilayer electrolyte was developed using a cosputtering process. The cosputtered BHYb82 bilayer is dense and epitaxial with a composition of BaHf_0.83_Yb_0.17_O_3−δ_, which was shown to be well adhered and continuous across the uneven topography of a sintered half-cell. The optimized bilayer electrolytes displayed excellent chemical stability in harsh conditions such as high concentrations of CO_2_, with no detectable formation of BaCO_3_ after exposure to 100% CO_2_ at 500 °C for 100 h, while the unmodified BZCYYb1711 heavily degraded. Additionally, adequate protection was achieved with as little as 55 nm of BHYb82 on top of BZCYYb1711, and the film thickness could potentially be reduced to 15 nm given film uniformity could be increased. The total resistance of the bilayer electrolytes remained stable for over 1000 h in CO_2_, degrading only 0.4 to 1.1%/1000 h as opposed to 5.1 to 7.0%/1000 h for the unmodified cell. Single cells based on the bilayer electrolyte with a configuration of PBCC|BHYb82|BZCYYb1711|Ni-BZCYYb1711 demonstrated excellent electrochemical performance, achieving 1.22 W cm^–2^ in the fuel cell mode and −1.86 A cm^–2^ at 1.3 V in the electrolysis mode at 600 °C, while maintaining excellent durability. Additionally, the BHYb82 layer did not increase the ohmic resistance as compared to cells without the electrolyte protection layer, while greatly increasing the chemical stability and circumventing the traditional trade-off between conductivity and stability. This work demonstrates the successful application of a bilayer electrolyte and provides insights into increasing the stability and effectiveness of protective films for solid oxide electrolytes.

## Supporting Information Available

Experimental details, SEM images, XRD graphs, and additional data (PDF)
