# Supplementary material for: Durable and High-Performance Thin-Film BHYb-Coated BZCYYb Bilayer Electrolytes for Proton-Conducting Reversible Solid Oxide Cells
Source: ACS Appl Mater Interfaces. 2023 Jun 28;15(27):32395–403. doi: 10.1021/acsami.3c04627 (PMC10347428; doi:10.1021/acsami.3c04627)
Supplement: Supplementary file 1 — am3c04627_si_001.pdf [file am3c04627_si_001.pdf]

Supporting Information

**Durable and High-Performance Thin-Film BHYb Coated BZCYYb Bilayer Electrolytes for Proton-Conducting Reversible Solid Oxide Cells**

*Nicholas Kane,‡[a] Zheyu Luo,‡[a] Yucun Zhou,*[a] Yong Ding,[a] Alex Weidenbach,[b] Weilin Zhang,[a] and Meilin Liu*[a]*

[a]School of Materials Science and Engineering, Georgia Institute of Technology, 771 Ferst Dr. NW, Atlanta, GA, 30332-0245, USA.

E-mail: meilin.liu@mse.gatech.edu and yucun.zhou@mse.gatech.edu

[b]School of Electrical and Computer Engineering, Georgia Institute of Technology, 777 Atlantic Dr. NW, Atlanta, GA 30332-0250, USA.

# Experimental Methods

## Fabrication of Electrolyte Powers

BZCYYb1711 and BHYb82 electrolyte powders were fabricated *via* the solid-state reaction method. Stoichiometric amounts of $BaCO_3$, $ZrO_2$, $CeO_2$, $HfO_2$, $Y_2O_3$, and $Yb_2O_3$ were ball milled in ethanol to thoroughly mix the powders. The dried powder was then pressed into a large pellet and fired at 1100 °C. The fired pellet was crushed into a power and the process (ball milling, pressing, annealing) was repeated until desired phase is formed. Finally, the powder was ball milled with PVB (binder) and NiO (sintering aid) before pressing into pellets and sintering at 1400 °C for 5 h. To measure conductivity, Ag electrodes were pasted to both sides of the pellets and fired at 800 °C for 1 h.

## Fabrication of Single Cells

BZCYYb-based fuel electrode-supported half cells were fabricated with a configuration of NiO-BZCYYb|BZCYYb via co-tape casting and co-sintering. For the fuel electrode, BZCYYb

powder and NiO powder were mixed in a 4:6 ratio by weight. To form the slurries, the power was mixed with a dispersing agent, binder, plasticizer, and pore former in ethanol. The slurries were then tape cast on a Mylar film, with the electrolyte layer first, followed by the fuel electrode functional layer, and the fuel electrode supporting layer. The tri-layer tapes were then fired at 1400 °C for 5 h. After firing, the fuel electrode layer was flattened and polished with SiC polishing paper up to 1200 grit to increase thermal conduction between the sputtering stage and the cell. After film deposition, a $PrBa_{0.8}Ca_{0.2}Co_2O_{5+\delta}$ (PBCC) air electrode with an area of 0.28 $cm^2$ was screen printed on the BHYb82 layer using PBCC paste (a mixture of PBCC powder and terpineol (5 wt% ethyl cellulose)) and fired at 950 °C for 2 h. The PBCC powder was synthesized using a sol-gel method by dissolving stoichiometric amounts of $Pr(NO_3)_3*6H_2O$, $Ba(NO_3)_2$, $Ca(NO_3)_2*4H_2O$, and $Co(NO_3)_2*6H_2O$ in distilled water with proper amounts of ethylene glycol and anhydrous citric acid (1:1 ratio) and followed by heating to 350 °C in air and combustion to form powder. The resulting powder was then ground and calcined at 900 °C for 2 h.

**Deposition of BHYb82 Films**

The BHYb82 layer of the bilayer electrolyte was fabricated via co-sputtering BHYb82 and Ba targets using the system shown in Figure S20. The BHYb82 target powder was fabricated *via* solid-state reaction, as previously described. To fabricate the target, BHYb82 powder was mixed with PVB and NiO and pressed in a 30 mm die. The pellet was fired at 1450 °C for 5 h. To fabricate the Ba target, a 1 cm segment was cut inside an Ar glove box from a Ba metal rod (99.5% pure) measuring approximately 2 cm x 20 cm (Alfa Aesar). The 1 cm thick disk was then coated in mineral oil and pressed in a hydraulic press until the diameter measured slightly over 2.54 cm, with a thickness of approximately 0.32 cm. Finally, SiC polishing paper was used to reduce the

diameter to 2.54 cm, while continually adding mineral oil. The mineral oil was removed with isooctane before loading the target into the system.

Prior to deposition, the cells (fuel electrode supported half cells or electrolyte pellets) were cleaned in an $O_2$ plasma cleaner (Harrick PDC-001) for at least 15 minutes. The deposition chamber was pumped down to $10^{-7}$ mTorr and backfilled with Ar at 15 sccm to achieve an operating pressure of 5 mTorr. The BHYb82 target was sputtered with a 13.56 MHz RF magnetron sputtering gun operating at 4 W $cm^{-2}$. The Ba metal target was driven with a DC source operating at 0.6 to 1.2 W $cm^{-2}$. A 304 stainless steel mesh (40x40 mesh size, 11 mil wire diameter, 31% open area) was placed approximately 5 cm below the Ba target to reduce the deposition rate of the Ba, as shown in Figure S21. The stage temperature was set at 650 °C. Post deposition annealing was performed at 950 °C for 2 h, which is the annealing procedure for the air electrodes used in this work. For cells with an air electrode, post deposition annealing was performed concurrently with the air electrode annealing.

**Stability Testing**

For the exposure to water or $CO_2$, samples were contained in a quartz tube. For high concentrations of water, a water bubbler was heated to the relevant temperature (e.g., room temperature for 3% $H_2O$ and 94 °C for 80% $H_2O$) using heat tape and a PID controller or a laboratory oven, with the gas lines from the bubbler to the cell fixture heated to at least 10 °C above the bubbler set point to prevent condensation. Ar carrier gas was controlled with a mass flow controller set at 10 to 30 sccm.

**Characterization**

Scanning electron microscopy was performed with a Hitachi SU8230 FE-SEM. Energy dispersive X-ray spectroscopy was performed with an Oxford Instruments X-Max$^N$ detector. X-

ray diffraction was performed with a Panalytical X'Pert PRO Alpha-1 diffractometer using Cu Kα1 radiation. Raman spectroscopy was performed with a Renishaw RM1000 operating with a 514 nm laser excitation. For TEM analysis, the cross-section was cut from the bulk sample using a Thermal Helios 5 CX focused ion beam. After cross-sectioning, the sample was imaged with a FEI Tecnai F30 TEM operating at 300 kV.

For transference number testing, the symmetrical cells were bonded to an alumina support tube with fritted glass paste, which was composed of GM31107 glass powder (Schott, Germany) and 3.3 wt% ethyl cellulose in terpineol in a 6:1 weight ratio. The mounted cell was annealed at 700 °C for 1 hour before testing. 20 sccm $H_2$ (3% $H_2O$) was introduced to the fuel side and air (3% $H_2O$) was introduced to the air side. The voltage of the concentration cell was measured with a Keithley 2700 Multimeter.

For electrochemical measurements, single cells were mounted on an alumina support tube with Ceramabond 552 (Aremco). In the fuel cell mode, 20 sccm humidified $H_2$ (3% $H_2O$) was supplied to the fuel electrode as the fuel and ambient air in the air electrode was used as the oxidant. In the electrolysis mode, 20 sccm humidified $H_2$ (3% $H_2O$) was supplied to the fuel electrode and 100 sccm humidified air (3% to 50% $H_2O$) was produced using the previously described bubbler setup and supplied to the air electrode. Impedance spectra were acquired over a frequency range of 0.1 Hz to 100 kHz with an AC amplitude of 20 mV using a Princeton Applied Research Parstat MC. The I-V curves of cells were acquired using an Arbin multi-channel electrochemical testing system.

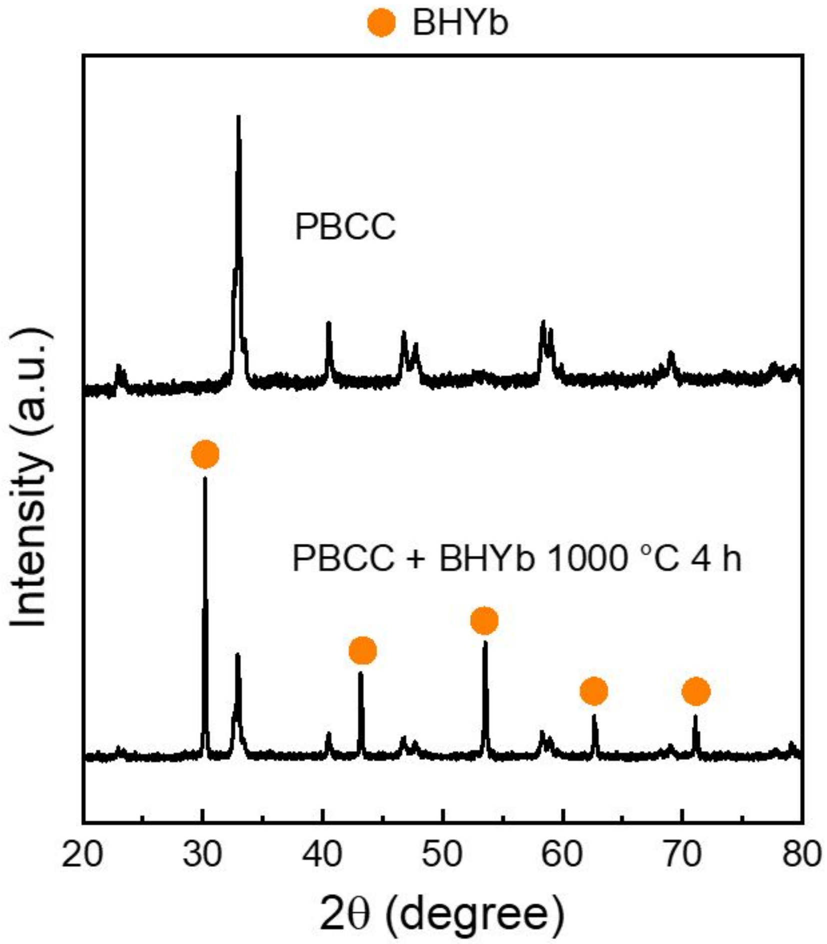


Figure S1. XRD patterns of PBCC and a mixture of PBCC and BHYb82 fired at 1000 °C for 4 h.

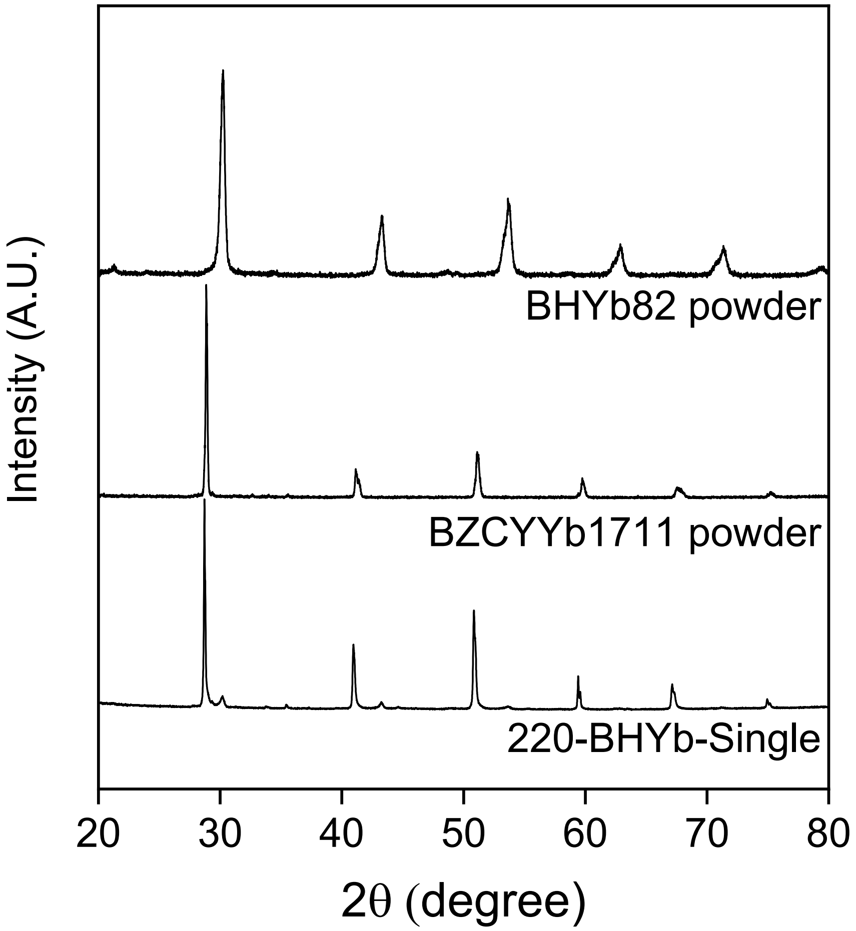


Figure S2. XRD patterns of BHYb82 and BZCYYb1711 references and a 220 nm single target BHYb82 film on BZCYYb1711 with a deposition temperature of 500 °C and annealing temperature of 1300 °C.

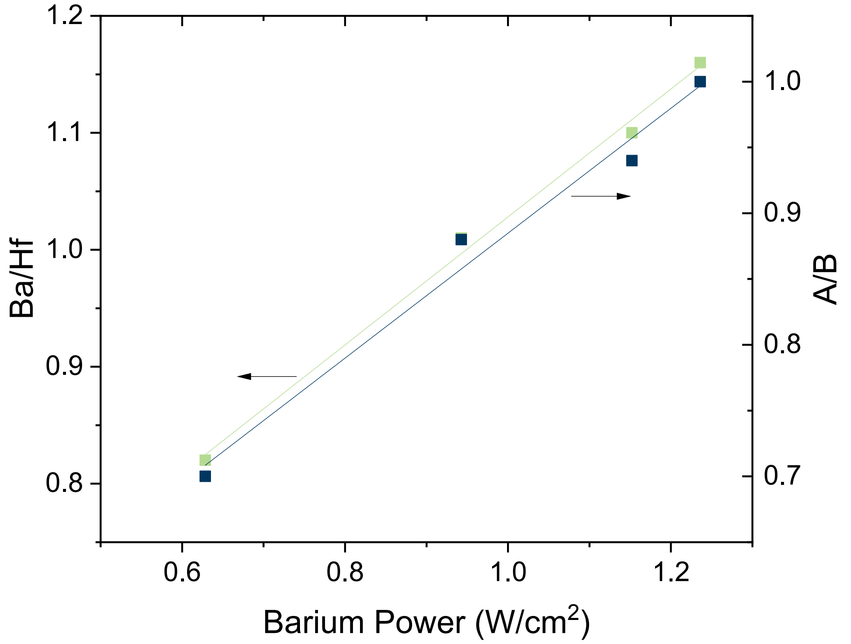


Figure S3. Barium to hafnium and A-site to B-site ratios as a function of barium target power.

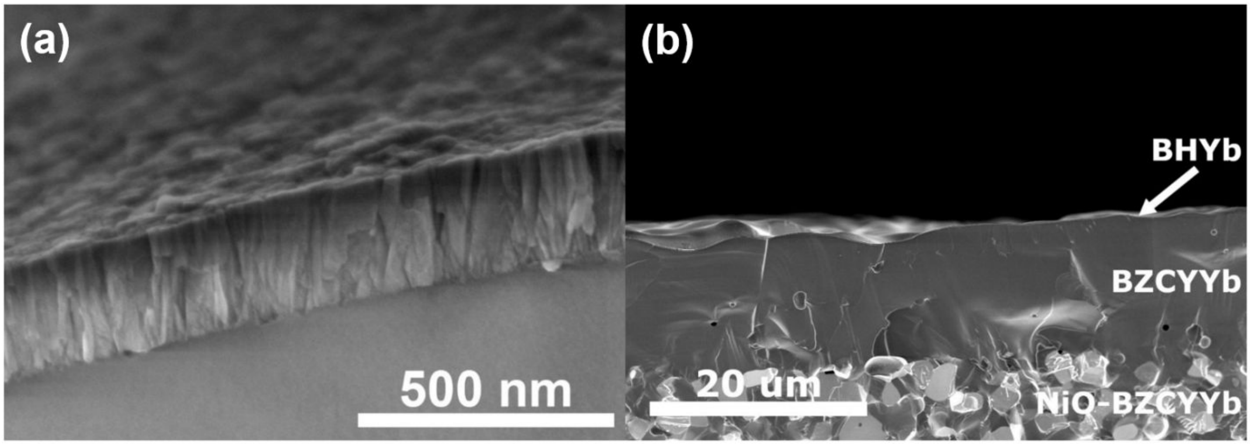


Figure S4. Cross-sectional SEM images of the BHYb-BZCYYb bilayer electrolyte. (a) High magnification image of 220-BHYb. (b) Low magnification image of 220-BHYb showing the entire electrolyte cross section.

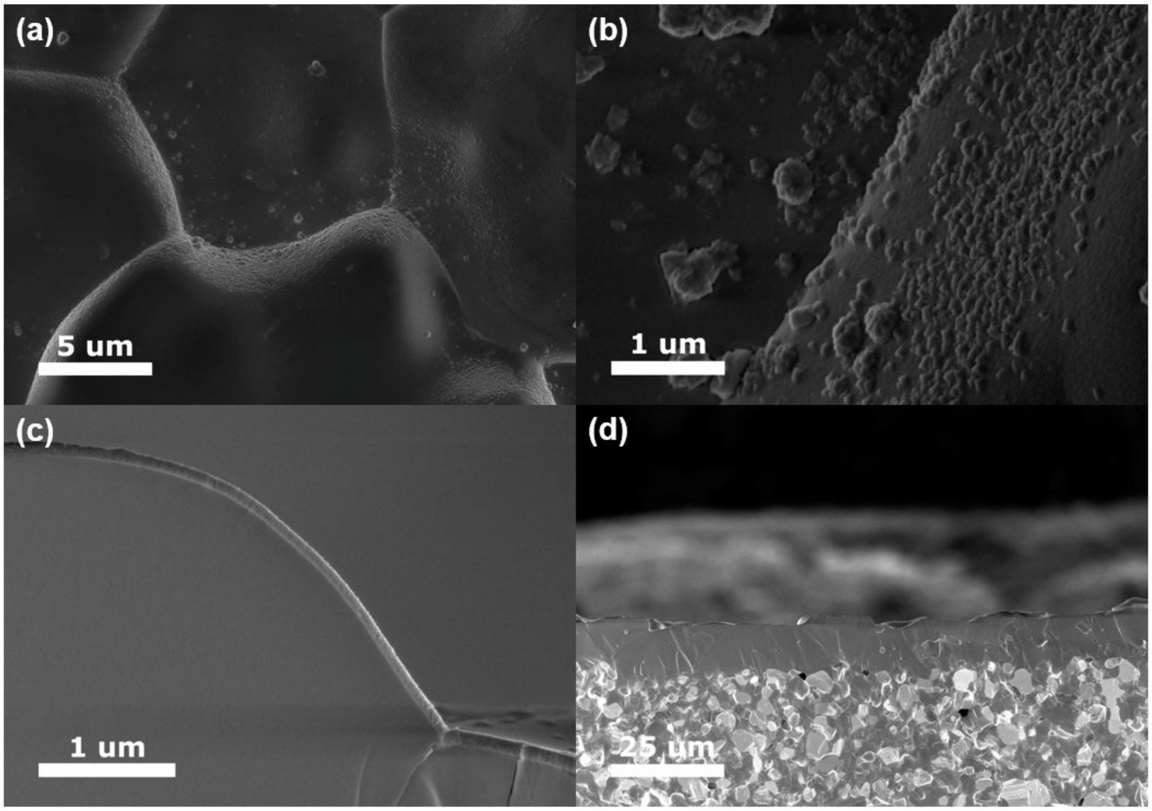


Figure S5. SEM images of the (a-b) surface and (c-d) cross-section of the BHYb-BZCYYb bilayer electrolytes showing faceting of the film around euneven surfaces.

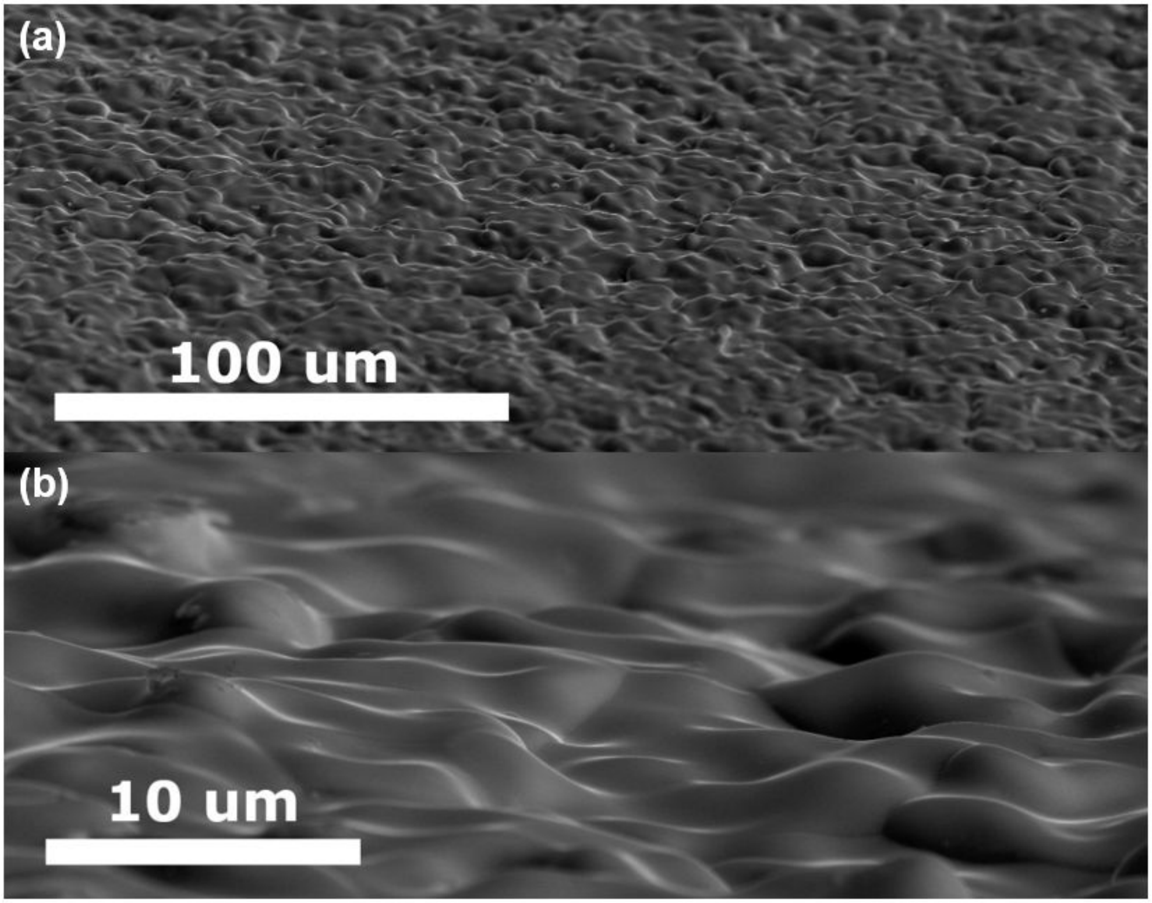


Figure S6. (a-b) SEM images of the surface of a BZCYYb half-cell from a glancing angle to highlight the rough topography of the surface.

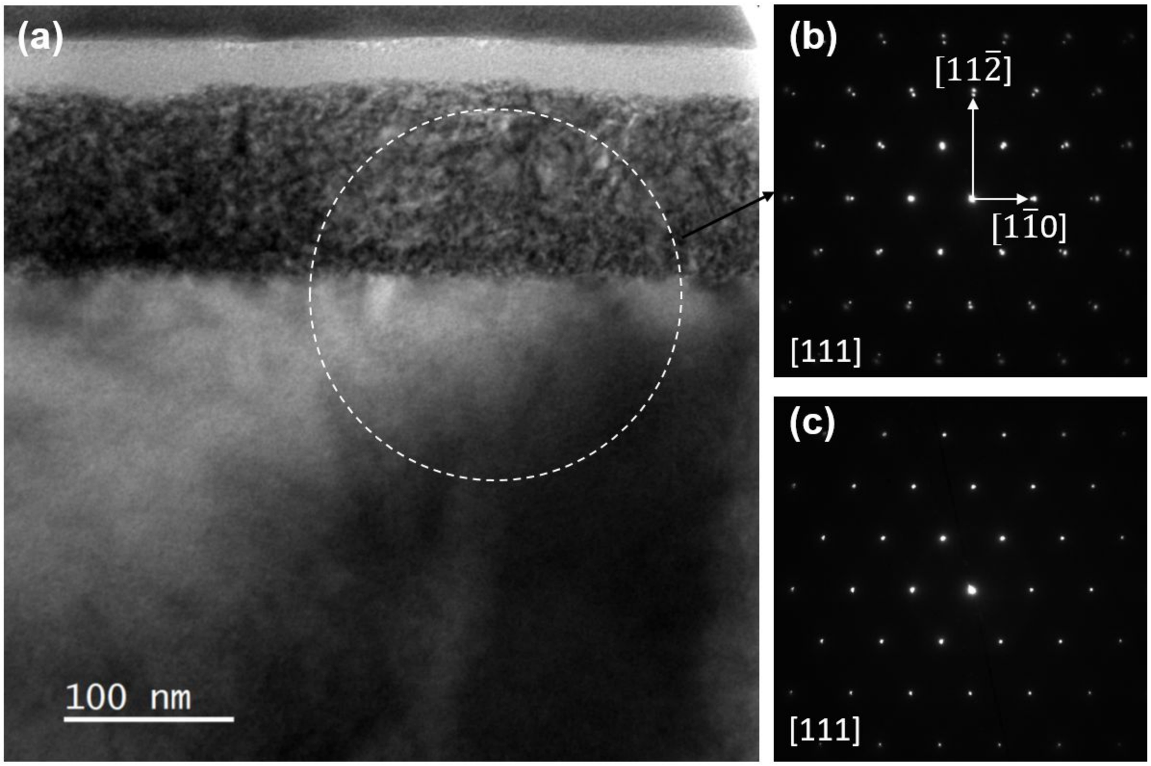


Figure S7. Additional TEM analysis of the 110-BHYb bilayer electrolytes. (a) STEM image of the surface of the film. (b) SAED pattern of the BHYb82 film and BZCYYb1711 substrate. (c) SAED pattern of the BZCYYb1711 substrate.

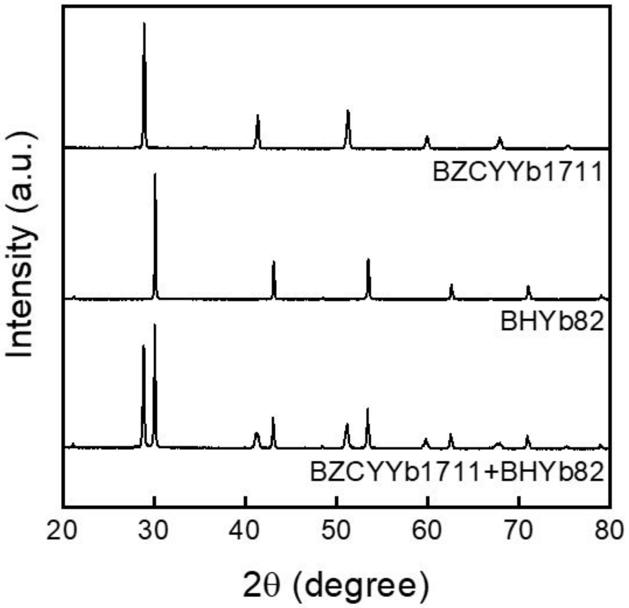


Figure S8. XRD patterns of BZCYYb1711 and BHYb82 pellets and the mixture between BZCYYb1711 and BHYb82 fired at 1000 °C for 4 h.

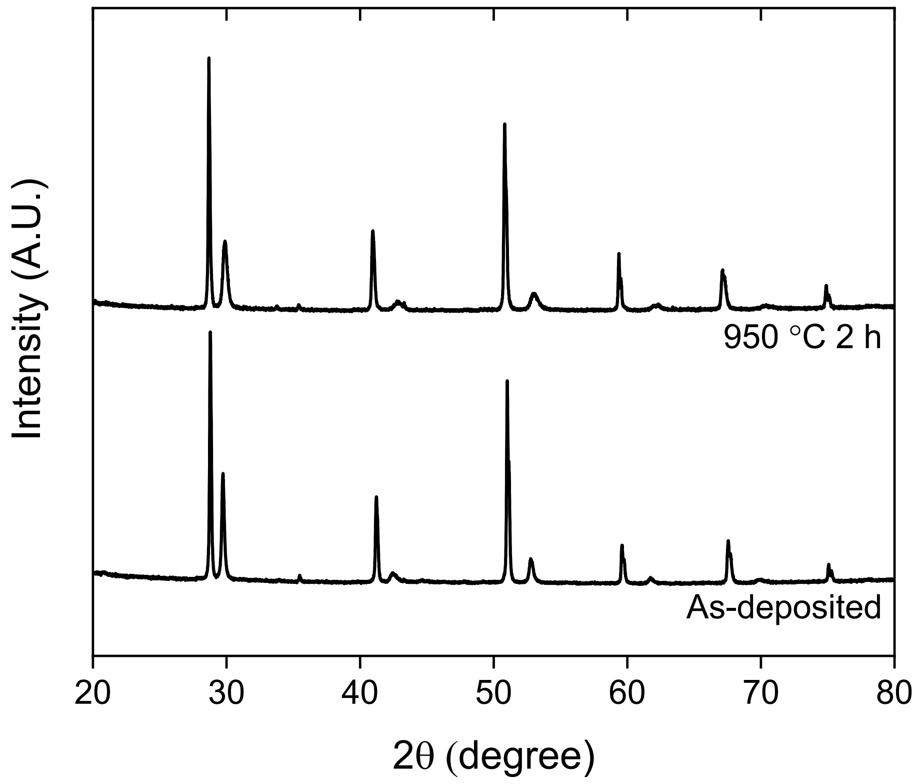


Figure S9. XRD patterns of the as-deposited and annealed BHYb-BZCYYb bilayers.

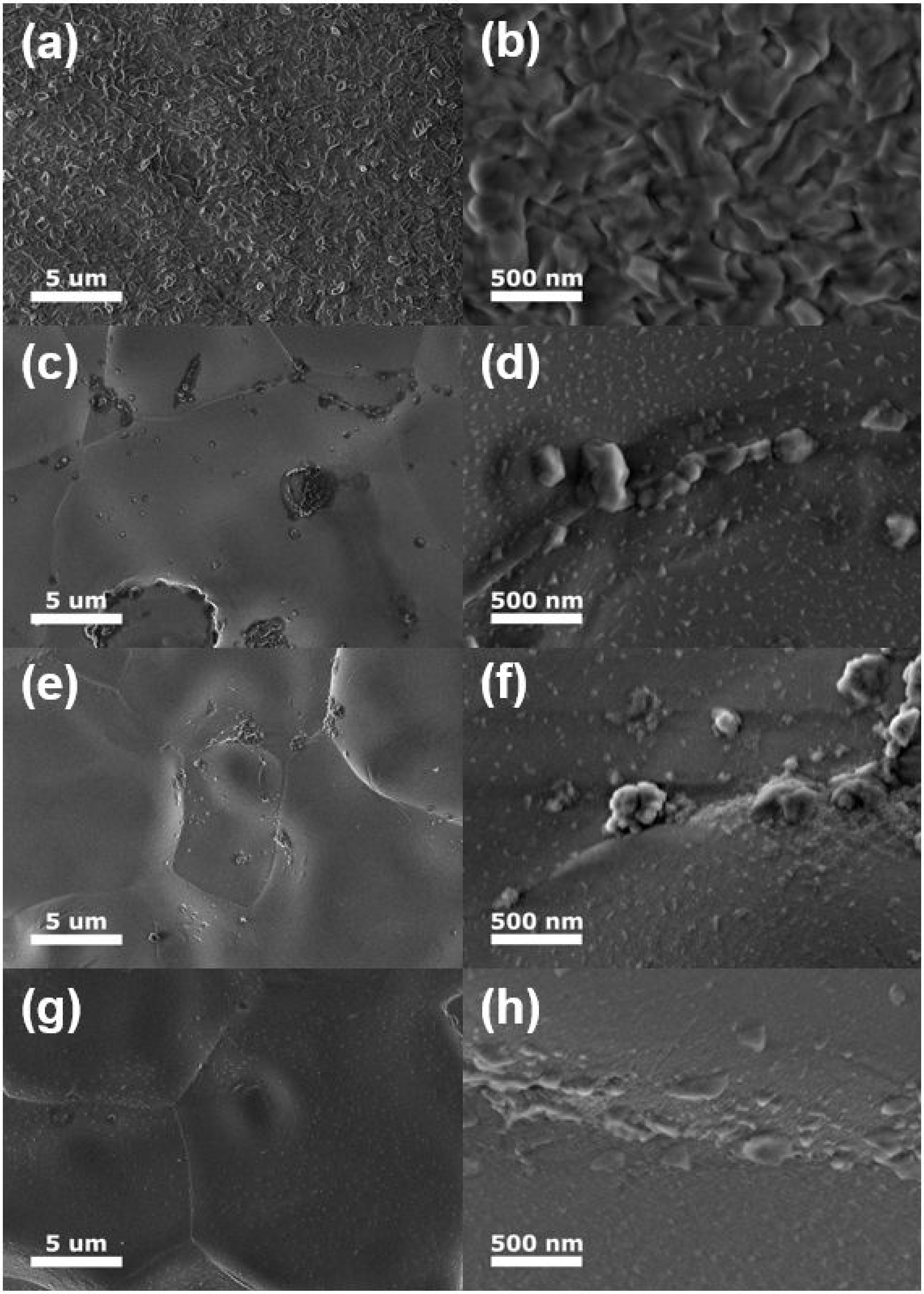


Figure S10. Additional SEM images of the 100-hour 100% $CO_2$ stability test at 500 °C for (a-b) bare BZCYYb1711, (c-d) 15-BHYb, (e-f) 55-BHYb, and (h-g) 110-BHYb.

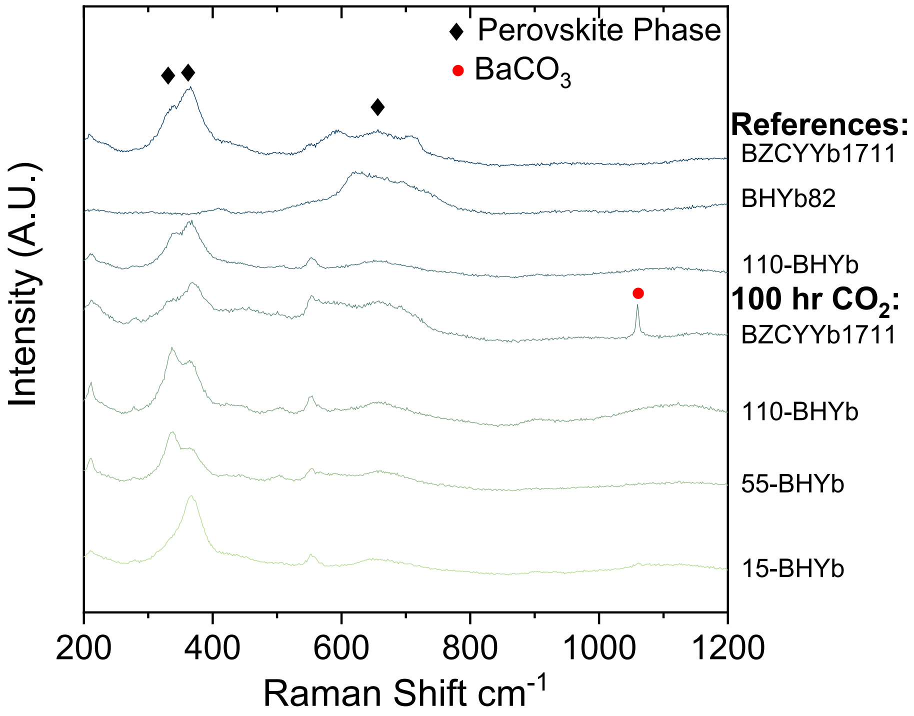


Figure S11. Raman spectra of the BZCYYb1711, BHYb82, and BHYb-BZCYYb bilayers before and after stability testing for 100 hours in 100% $CO_2$ at 500 °C.

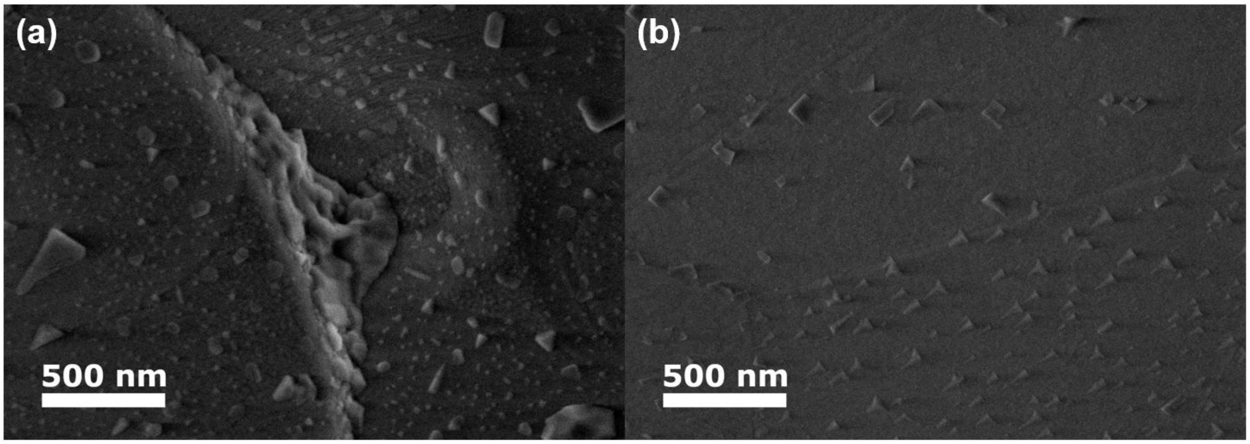


Figure S12. SEM images of 110-BHYb on a (a) as-sintered and (b) polished BZCYYb surface after stability testing in 97% $CO_2$ with 3% $H_2O$ for 1000 h at 500 °C.

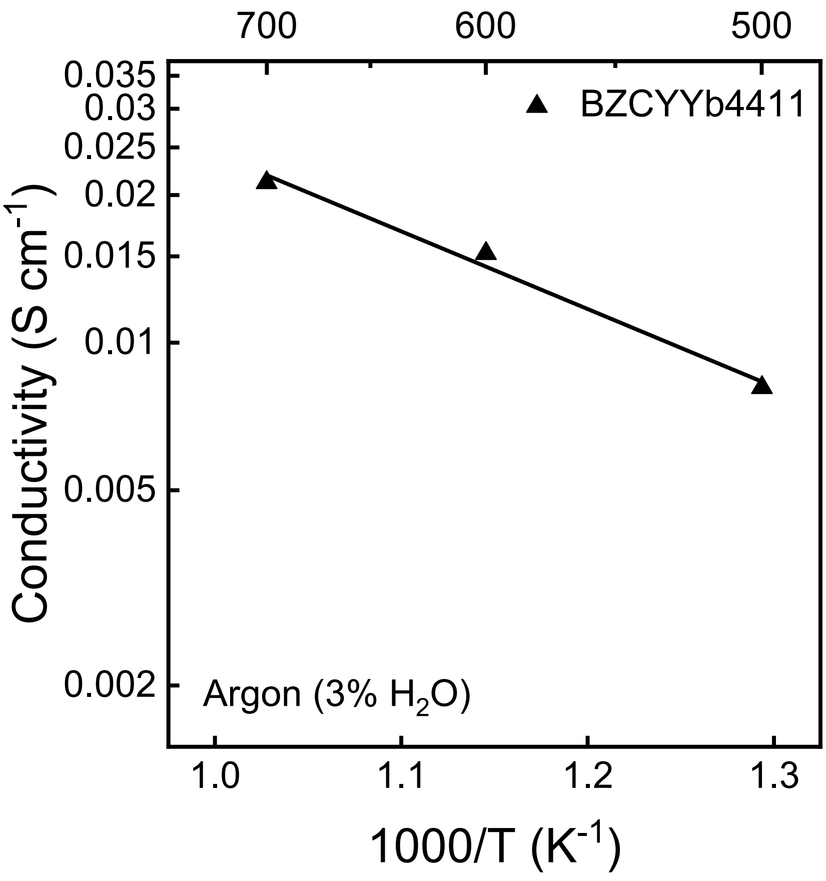


Figure S13. Conductivity of BZCYYb4411 as a function of temperature from 500 to 700 °C.

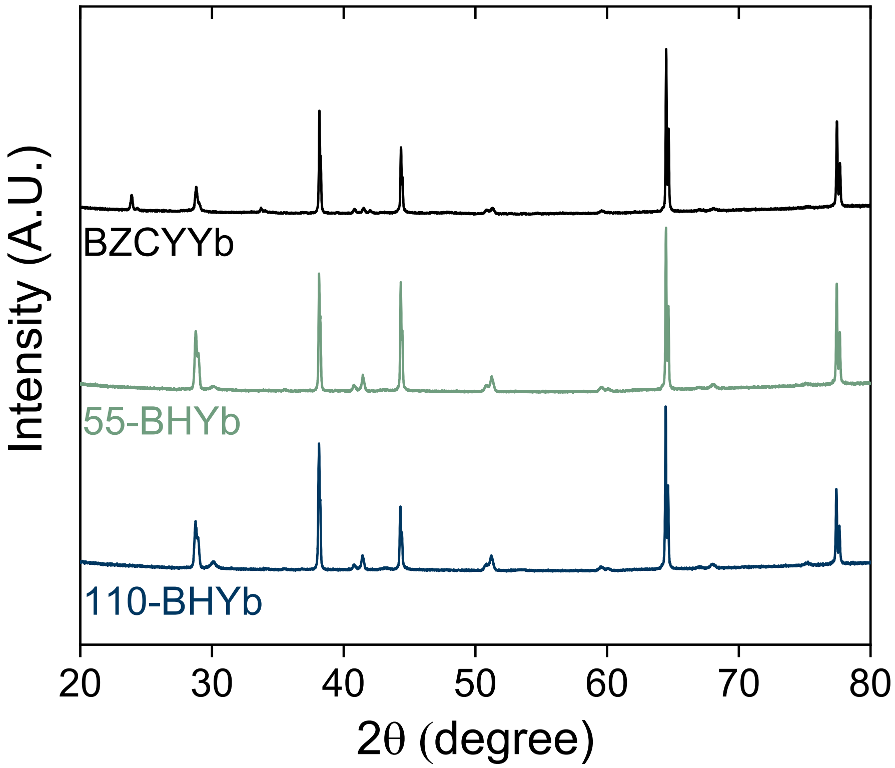


Figure S14. XRD patterns of the bare BZCYYb1711, 55-BHYb, and 110-BHYb electrolytes after exposure to 97% $CO_2$ with 3% $H_2O$ at 500 °C for 1000 h.

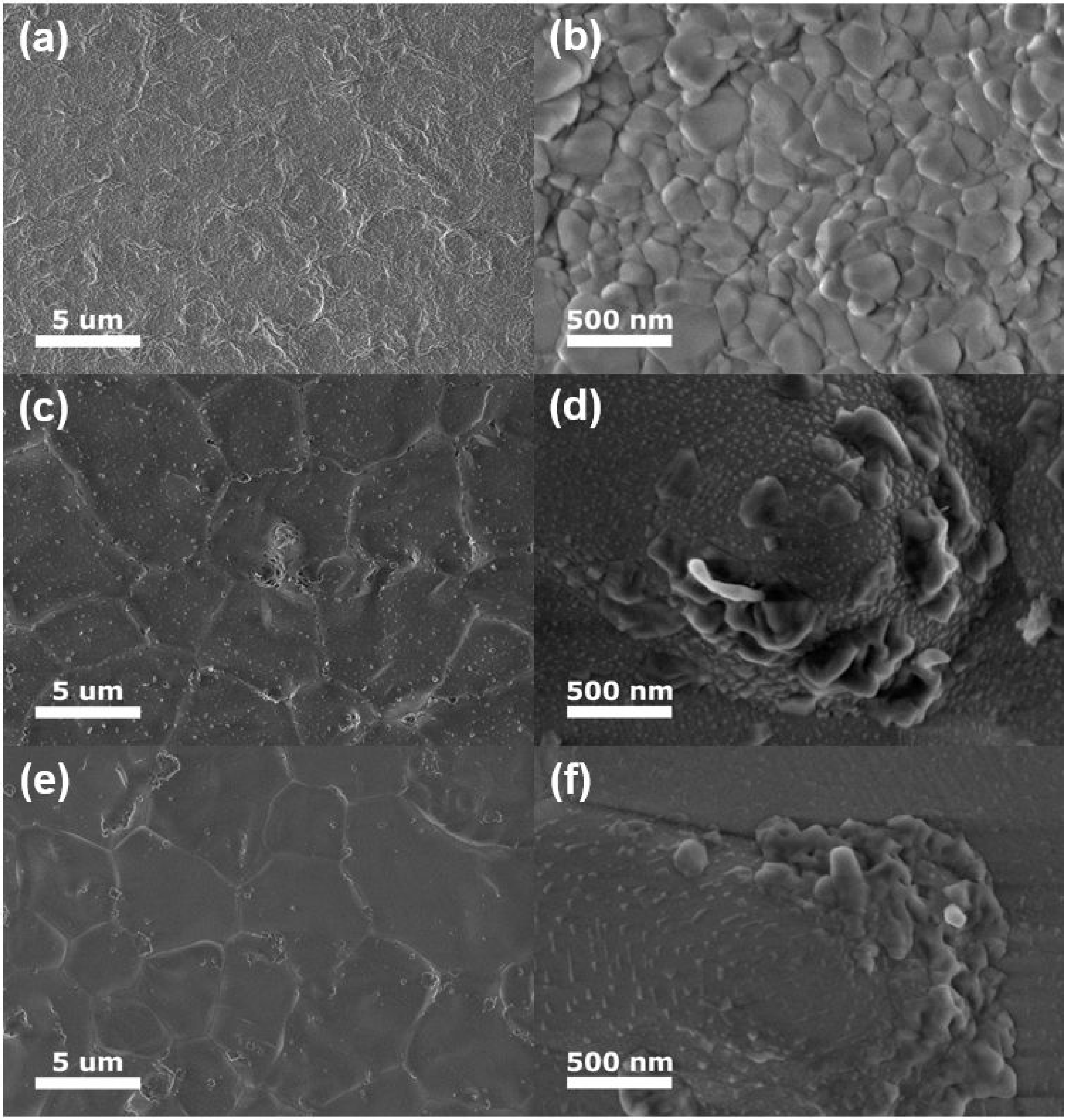


Figure S15. SEM images of (a-b) bare BZCYYb1711, (c-d) 55-BHYb, and (e-f) 110-BHYb after the 1000 h stability test in 97% $CO_2$ with 3% $H_2O$ at 500 °C.

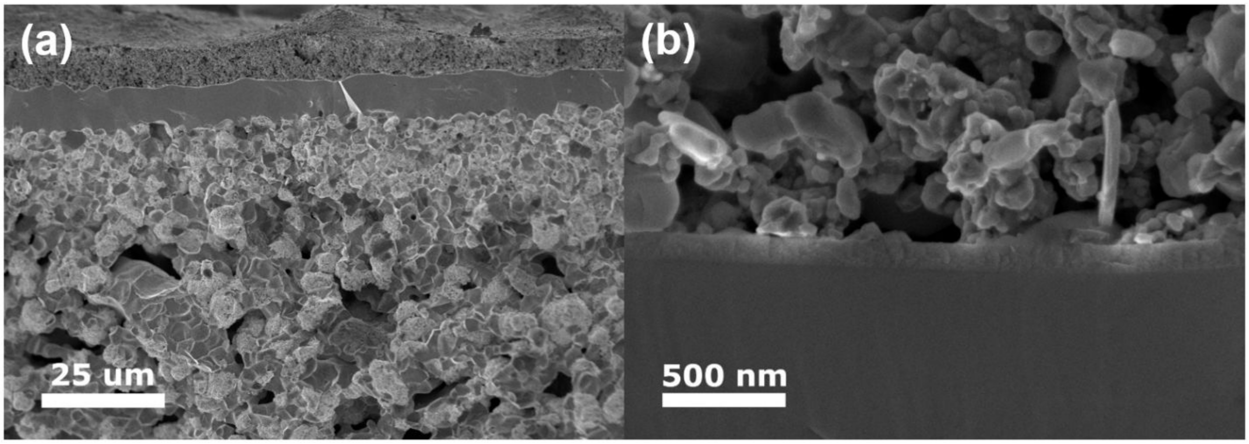


Figure S16. (a-b) SEM images of the 110-BHYb bilayer-based single cells after electrode firing at 950 °C for 2 h.

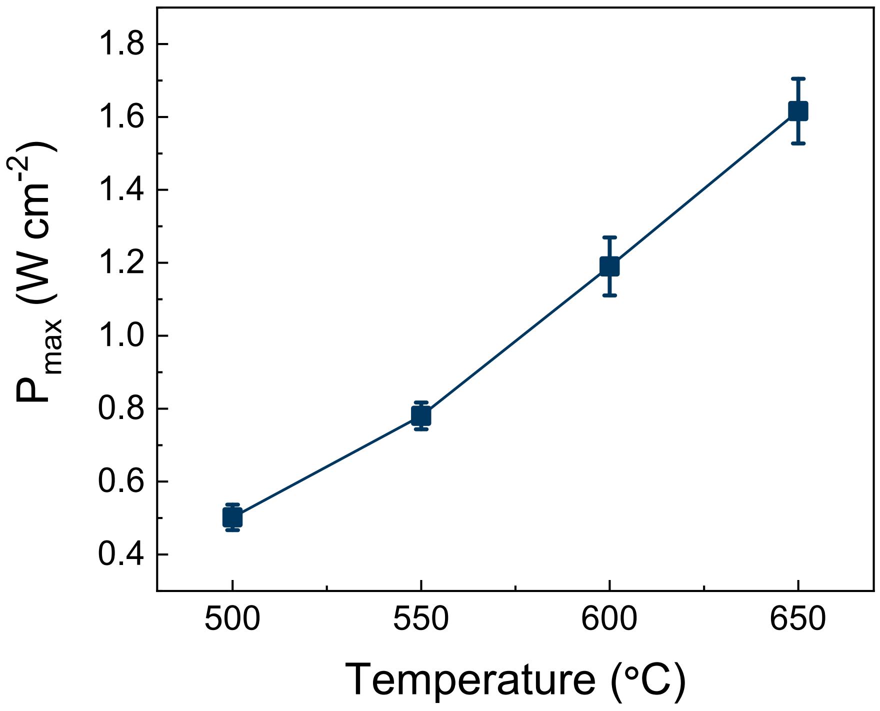


Figure S17. The average peak power density of five 110-BHYb based single cells with error bars showing the reproducibility.

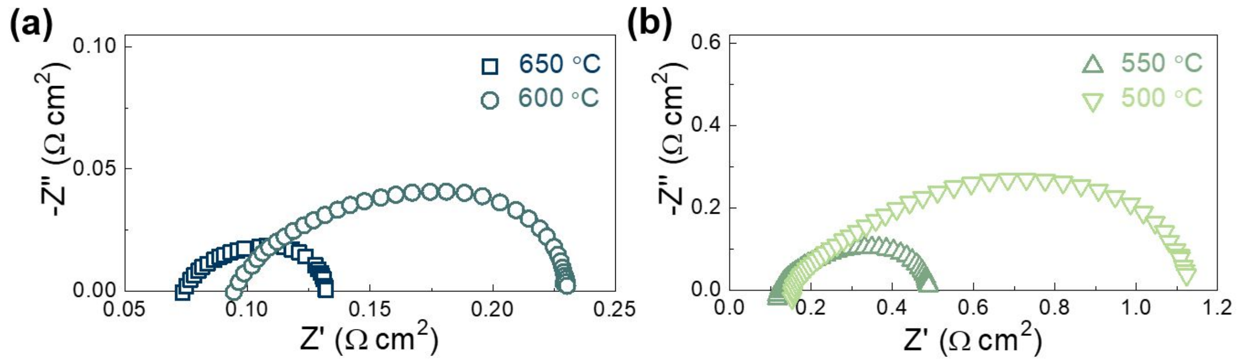


Figure S18. Nyquist plots of the 110-BHYb based single cells with $H_2$ (3% $H_2O$) on the fuel side and air (3% $H_2O$) on the air side at (a) 650 and 600 °C and (b) 550 and 500 °C.

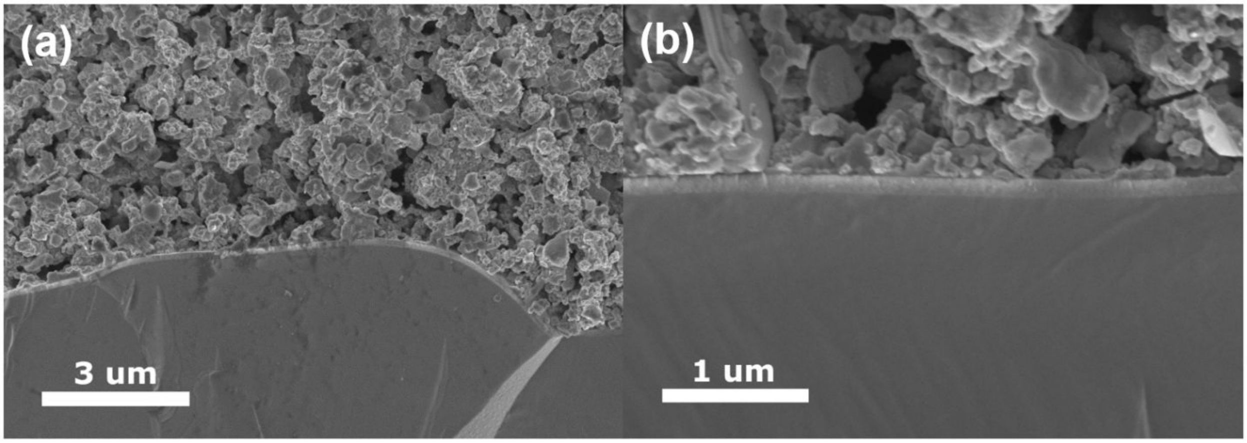


Figure S19. (a-b) SEM images of the 110-BHYb bilayer-based single cells after 500 h stability testing at 600 °C with $H_2$ (3% $H_2O$) on the fuel side and air (3% $H_2O$) on the air side.

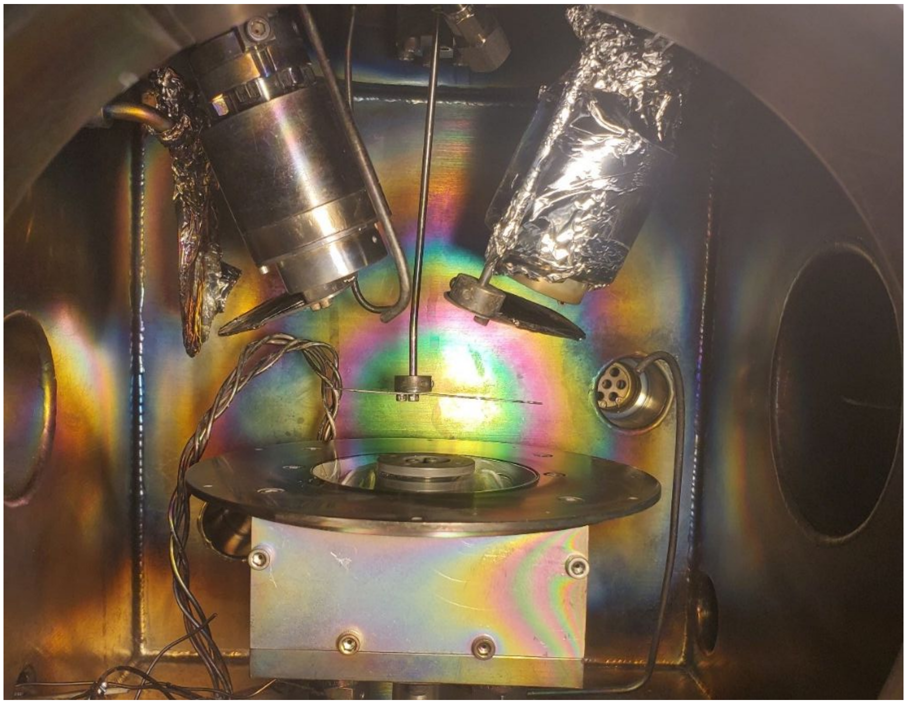
Figure S20. Image of the co-sputtering system.

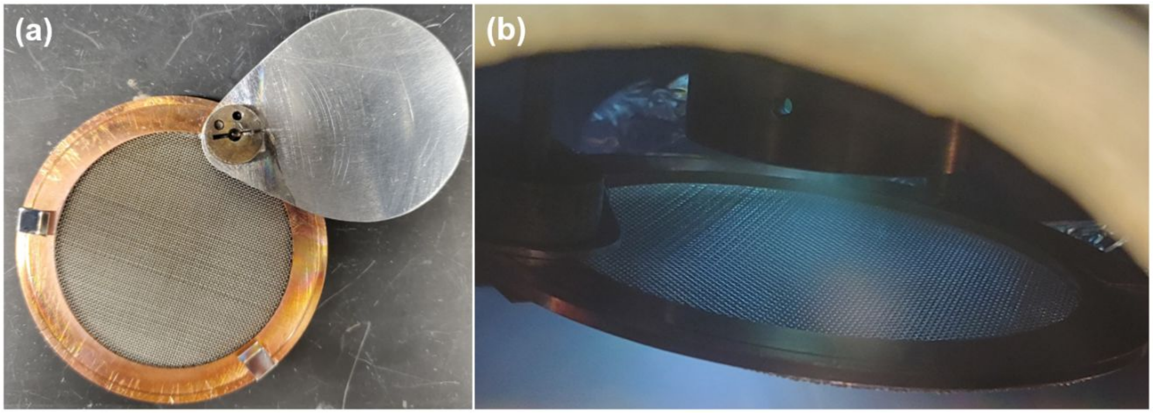


Figure S21. Images of the shutter and mesh assembly used to decrease the deposition rate of barium during the co-sputtering process. (a) Outside the chamber and (b) in operation.

Table S1. Composition of the single target BHYb thick film on a Si substrate measured via EDX, showing severe Ba deficiency.

| Element | Atomic % |
|---|---|
| Ba | 13.46 |
| Hf | 19.81 |
| Yb | 4.85 |
| O | 61.27 |
| Ba:Hf | 0.68 |
| A:B | 0.55 |

Table S2. Composition of the co-sputtered BHYb thin film on a Ag substrate measured via EDX.

| **Element** | **Atomic %** |
|---|---|
| Ba | 6.05 |
| Hf | 5.01 |
| Yb | 1.06 |
| Ag | 23.29 |
| O | 64.03 |
| Ba:Hf | 1.21 |
| A:B | 1.00 |
